# Merits of photocatalytic and antimicrobial applications of gamma-irradiated Co_*x*_Ni_1−*x*_Fe_2_O_4_/SiO_2_/TiO_2_; *x* = 0.9 nanocomposite for pyridine removal and pathogenic bacteria/fungi disinfection: implication for wastewater treatment†

**DOI:** 10.1039/c9ra10505k

**Published:** 2020-02-03

**Authors:** Gharieb S. El-Sayyad, M. Abd Elkodous, Ahmed M. El-Khawaga, Mohamed A. Elsayed, Ahmed I. El-Batal, Mohamed Gobara

**Affiliations:** Drug Microbiology Laboratory, Drug Radiation Research Department, National Center for Radiation Research and Technology (NCRRT), Egyptian Atomic Energy Authority Cairo Egypt Gharieb.Elsayyad@eaea.org.eg; Department of Electrical and Electronic Information Engineering, Toyohashi University of Technology Toyohashi Aichi 441-8580 Japan mohamed.hamada.abdlekodous.xi@tut.jp; Center for Nanotechnology (CNT), School of Engineering and Applied Sciences, Nile University Sheikh Zayed Giza 16453 Egypt; Chemical Engineering Department, Military Technical College, Egyptian Armed Forces Cairo Egypt

## Abstract

In this paper, we report a layer-by-layer approach for the preparation of a concentric recyclable composite (Co_*x*_Ni_1−*x*_Fe_2_O_4_/SiO_2_/TiO_2_; *x* = 0.9) designed for wastewater treatment. The prepared composite was investigated by X-ray diffraction spectroscopy, high-resolution transmission electron microscopy and scanning electron microscopy (SEM) supported with energy dispersive X-ray (EDX) spectroscopy to analyze crystallinity, average particle size, morphology and elemental composition, respectively. The antimicrobial activities of the prepared composite have been investigated against multi-drug-resistant bacteria and pathogenic fungi using a variety of experiments, such as zone of inhibition, minimum inhibitory concentration, biofilm formation and SEM with EDX analysis of the treated bacterial cells. In addition, the effects of gamma irradiation (with different doses) and UV irradiation on the antibacterial abilities of the prepared composite have been evaluated. Moreover, the effect of gamma irradiation on the crystallite size of the prepared composite has been studied under varying doses of radiation (25 kGy, 50 kGy and 100 kGy). Finally, the photocatalytic efficiency of the prepared composite was tested for halogen-lamp-assisted removal of pyridine (artificial wastewater). Various parameters affecting the efficiency of the photocatalytic degradation, such as photocatalyst dose, pyridine concentration, pH, point of zero charge and the presence of hydrogen peroxide, have been studied. Our results show that the synthesized composite has a well-crystallized semi-spherical morphology with an average particle size of 125.84 nm. In addition, it possesses a high degree of purity, as revealed by EDX elemental analysis. Interestingly, the prepared composite showed promising antibacterial abilities against almost all the tested pathogenic bacteria and unicellular fungi, and this was further improved after gamma and UV irradiation. Finally, the prepared composite was very efficient in the light-assisted degradation of pyridine and its degradation efficiency can be tuned based on various experimental parameters. This work provides a revolutionary nanomaterial-based solution for the global water shortage and water contamination by offering a new wastewater treatment technique that is recyclable, cost effective and has an acceptable time and quality of water.

## Abstract 2

In this paper, we report a layer-by-layer approach for the preparation of a concentric recyclable composite (Co_*x*_Ni_1−*x*_Fe_2_O_4_/SiO_2_/TiO_2_; *x* = 0.9) designed for wastewater treatment.

## Graphical abstract

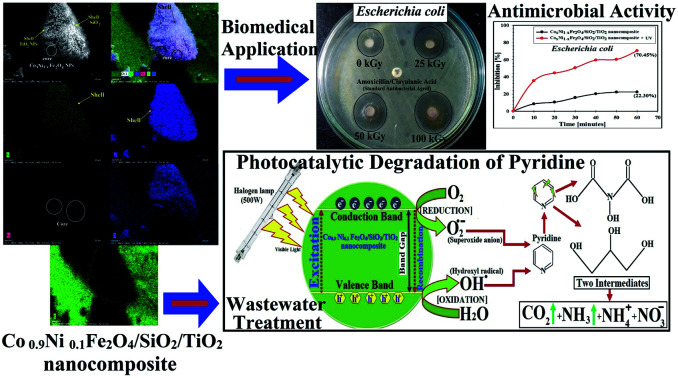

## 1 Introduction

Water contamination is one of the most serious factors affecting public health and terrestrial and aquatic environments.^1^ In addition, the percentage of available potable water on Earth, on which all living organisms rely, is tiny (only about 1%).^2^ Moreover, 6–8 million people die every year due to water-borne pathogens, which cause serious diseases, such as typhoid fever, diarrhea and hepatitis A.^3–5^ Wastewater contains different kinds of pollutants, such as heavy metals, dissolved and non-dissolved chemicals, phenols, dyes and other miscellaneous substances.^6,7^

Among these pollutants, pyridine receives continuous attention due to the severity of its effects.^8^ Pyridine (C_5_H_5_N) is an organic compound with a basic heterocyclic structure.^9,10^ It is a colorless, volatile and flammable liquid possessing an unpleasant odor and high toxicity.^11^ Pyridine exposure has critical effects on the human immune system and may lead to carcinogenicity.^12^ In addition, pyridine derivatives exhibit toxicity to aquatic life and produce irritation due to their unpleasant smell.^13^ Currently, more than 25 000 tons per year of pyridine derivatives are produced worldwide because of their widespread use as herbicides and insecticides in cultivation and in some industrial activities, including textile manufacture, chemical and pharmaceutical synthesis.^14^

Thus, wastewater treatment and strategies for water preservation should be a global consideration. Chemical and microbial removal of various kinds of pollutants are currently employed in traditional wastewater treatment plants.^15^ However, the efficiency of degradation of pollutants, the capacity of wastewater treatment plants and the treatment time all have serious limitations. To overcome these limitations more effective and satisfactory techniques for wastewater treatment are required. Advances in nanotechnology using nanomaterials provides a revolutionary solution for these issues and can also improve the efficacy of traditional wastewater treatment plants.^16^

Nanomaterials possess relatively higher degrees of chemical, physical and biological activities due to their large surface area with respect to their bulk counterparts.^17–21^ Among nanomaterials, titanium dioxide (TiO_2_) is still a very promising candidate for light-assisted photocatalysis and degradation of many kinds of pollutant present in wastewater.^22^

TiO_2_ nanoparticles (NPs) are chemically active, abundant, non-toxic and possess satisfactory photocatalytic activity.^23^ However, nanoparticles are too small to adsorb large quantities of pollutants and collection and reusability of the particles are very important considerations in terms of the overall cost. Thus, designing efficient and reusable photocatalytic systems for wastewater treatment based on nanomaterials is of critical importance.

Herein, we report the preparation of an improved recyclable nanocomposite (Co_*x*_Ni_1−*x*_Fe_2_O_4_/SiO_2_/TiO_2_; *x* = 0.9) synthesized by a layer-by-layer approach. The Co_*x*_Ni_1−*x*_Fe_2_O_4_; *x* = 0.9 NPs are employed as a magnetic core for reusability (Fig. S1†) and easy collection of the material after successive wastewater treatment operations, while the SiO_2_ layer was used to separate the magnetic core from the photoactive TiO_2_ layer, so as not to reduce its quantum efficiency. Finally, the TiO_2_ layer was used as a photoactive catalyst for the removal of pollutants present in wastewater. The antimicrobial activities of the prepared composite were tested against pathogenic bacteria and unicellular fungi, such as *Klebsiella pneumoniae*, *Proteus vulgaris*, *Proteus mirabilis*, *Salmonella typhi*, *Staphylococcus aureus*, *Escherichia coli*, *Pseudomonas aeruginosa*, *Bacillus subtilis*, *Candida tropicalis* and *Candida albicans*. In addition, the effect of both gamma (with different doses) and UV irradiation on the antimicrobial activities of the prepared composite have been analyzed. The effects of different doses of gamma irradiation on the crystallite size of the prepared composite have also been studied. Moreover, the photocatalytic ability of the prepared composite was tested for the degradation of pyridine solution (as an artificial wastewater). Different parameters controlling the photocatalytic efficiency, such as photocatalyst dose, pH, pyridine concentration and the presence of H_2_O_2_ have been studied.

## 2 Materials and methods

### 2.1. Materials

Tetraethyl orthosilicate (TEOS) 98% [Si(OC_2_H_5_)_4_], titanium(iv) isopropoxide 97% (C_12_H_28_O_4_Ti), ammonium hydroxide (NH_4_OH), nickel chloride (NiCl_2_), ferric chloride (FeCl_3_·6H_2_O), absolute ethanol (C_2_H_5_OH), ∼80 000 MW hydroxypropyl cellulose, sodium hydroxide (NaOH), cobalt chloride (CoCl_2_), and pyridine (C_5_H_5_N) were purchased from Sigma-Aldrich (Germany). Precursors for this study were used as received without further purification. Materials are commercially available and of extra-pure grade.

### 2.2. Methods

The nanocomposite under investigation was prepared and fully characterized according to the methods reported in our previous paper.^24^ We briefly present the preparation steps below and list the new experiments performed, such as new characterization data, degradation of pyridine, study of the parameters affecting degradation efficiency (photocatalyst dose, contaminant concentration, pH, presence of H_2_O_2_), antimicrobial activities against multi-drug-resistant bacteria and pathogenic fungi and the effect of gamma and UV irradiation on the antimicrobial activity of the prepared composite.

#### 2.2.1. Preparation of the core cobalt–nickel ferrite nanoparticles (Co_*x*_Ni_1−*x*_Fe_2_O_4_; *x* = 0.9)

Nanoparticles of Co_*x*_Ni_1−*x*_Fe_2_O_4_; *x* = 0.9 were prepared *via* a coprecipitation approach. First, nickel chloride (12.5 mg), cobalt chloride (12.5 mg) and ferric chloride 45% (0.05 ml) were dissolved in deionized (DI) water (50 ml) under slight heating to 80 °C. Then, the pH was raised to 8 by adding drops of aqueous sodium hydroxide solution (2 M) to the mixture, which lead to the formation and precipitation of Co_*x*_Ni_1−*x*_Fe_2_O_4_; *x* = 0.9 NPs. DI water was used to wash the obtained precipitate several times. Then, the precipitate was dried for 3 h at 70 °C. Finally, the powder was calcined at 300 °C for 4 h.

#### 2.2.2. Preparation of a core–shell structure (Co_*x*_Ni_1−*x*_Fe_2_O_4_; *x* = 0.9/SiO_2_)

First, Co_*x*_Ni_1−*x*_Fe_2_O_4_; *x* = 0.9 powder (180 mg) obtained as in Section 2.2.1 was dispersed in DI water (64 ml) *via* sonication for 45 min in an ultrasonic water bath. Then, ammonia solution (25%) (8 ml) and absolute ethanol (320 ml) were dropped directly into the mixture under constant stirring at room temperature. After that, TEOS (3.2 ml) was added drop-by-drop to the mixture, which was then left under constant stirring for 16 h. The precipitate was isolated by centrifugation and then washed with DI water and absolute ethanol many times. Finally, the precipitate was dried at 50 °C for 4 h.

#### 2.2.3. Preparation of the sandwich structure nanocomposite (Co_*x*_Ni_1−*x*_Fe_2_O_4_/SiO_2_/TiO_2_; *x* = 0.9)

First, Co_*x*_Ni_1−*x*_Fe_2_O_4_; *x* = 0.9/SiO_2_ powder obtained as in Section 2.2.2 was dispersed in 100 ml absolute ethanol, 0.2 g hydroxypropyl cellulose and 0.48 ml DI water with sonication for 30 min in an ultrasonic water bath. Then, titanium(iv) isopropoxide (4 ml) was dissolved in absolute ethanol (18 ml) in a separate vessel and was dripped directly into the mixture at a rate of 0.5 ml min^−1^. After that, the stirring speed was raised to 900 rpm and the temperature to 85 °C, and the reaction was left for 100 min under refluxing conditions. The precipitate produced was collected and washed with ethanol many times, after which it was redispersed in 20 ml of DI water. For partial etching of the silica layer and to form the hollow structure of the composite, the dispersion was mixed with 2 M NaOH solution (3.5 ml) under constant stirring for 1 h at room temperature. The powder was then collected, washed many times with DI water and dried at 90 °C for 4 h. Finally, the powder was calcined at 550 °C for 4 h.

#### 2.2.4. Characterization of the prepared nanocomposite

Phase, crystallinity and crystallite size were investigated by X-ray diffraction (XRD) spectroscopy using a Brucker Axis D8 diffractometer operating at 30 mA current, 40 kV voltage and using CuKα radiation (*λ* = 1.540598 Å). The average size of the particles was calculated using a JEM2100 (Jeol, Japan) high-resolution transmission electron microscope (HRTEM). Furthermore, the morphology, elemental composition and purity of the particles were analyzed using a Zeiss, EVO-MA10 scanning electron microscope (SEM) supported with an energy dispersive X-ray (EDX) spectroscopy unit (Bruker Nano GmbH D-12489, 410-M, Berlin, Germany). Finally, the zeta potential of the prepared composite at different pH values was measured using an ELS-Z1NT analyzer (Photal OTSUKA ELECTRONICS, Japan).

#### 2.2.5. Photocatalytic degradation of pyridine using Co_*x*_Ni_1−*x*_Fe_2_O_4_/SiO_2_/TiO_2_; *x* = 0.9 nanocomposite

The nanocomposite (10 mg) obtained as in Section 2.2.3 was added to 50 ml of an aqueous solution of pyridine with initial concentration *C*_0_ = 100 mg l^−1^, under constant stirring at ambient temperature (24.0 ± 2 °C) for 30 min in the dark, until adsorption–desorption equilibrium was attained between pyridine and the prepared photocatalyst (nanocomposite). After that a halogen lamp (500 W) was used as simulated visible light to irradiate the solution containing the photocatalyst and pyridine, which was axially located and held in a quartz immersion tube. At constant time intervals of irradiation, a syringe equipped with a filter (2.5 μm pore size) was used to draw out a sample of the pyridine suspension (1 ml). The sample was then centrifuged for 10 min at 5000 rpm to separate the photocatalyst.

The degradation rate of pyridine was calculated by determining the variation in pyridine concentration *versus* irradiation time using a UV-vis spectrophotometer (Agilent Technologies Cary 60 UV-vis) at *λ*_max_ = 256 nm. DI water was used as a ref. 25.

#### 2.2.6. Gamma irradiation

All prepared samples were gamma-irradiated at NCRRT, Cairo, Egypt. The irradiation source was the ^60^Co-Gamma Chamber 4000-A-India. The applied dose rate was fixed at 2.10 kGy h^−1^. Gamma irradiation displays hydrated and/or free radicals and solvated electrons after water radiolysis.^26^

#### 2.2.7. Antimicrobial activity of Co_*x*_Ni_1−*x*_Fe_2_O_4_/SiO_2_/TiO_2_; *x* = 0.9 nanocomposite

Each layer of the fabricated composite (the core Co_*x*_Ni_1−*x*_Fe_2_O_4_ NPs and the two shells of SiO_2_ and TiO_2_ NPs) and the full nanocomposite (Co_*x*_Ni_1−*x*_Fe_2_O_4_/SiO_2_/TiO_2_; *x* = 0.9) were dispersed in dimethyl sulfoxide (DMSO) to prepare a net concentration of 10 μg ml^−1^. Then, they were separately examined for their antimicrobial potential using the agar disc distribution technique.^27^ Additionally, the Co_*x*_Ni_1−*x*_Fe_2_O_4_/SiO_2_/TiO_2_; *x* = 0.9 nanocomposite was irradiated with gamma rays at various doses (25.0, 50.0 and 100.0 kGy) in order to investigate the effect on its antimicrobial activity.

All prepared samples (each layer of the composite and the whole nanocomposite) were examined against distinct isolates of pathogenic bacteria, such as *Staphylococcus aureus* (methicillin-resistant *S. aureus* (MRSA)), *Escherichia coli*, *Bacillus subtilis*, *Pseudomonas aeruginosa*, *Salmonella typhi*, *Klebsiella pneumoniae*, *Proteus vulgaris*, and *P. mirabilis*. In addition, the antifungal activity was examined against unicellular pathogenic fungi such as *Candida tropicalis* and *C. albicans*. The tested microorganisms were obtained from the culture collection of the Drug Microbiology Laboratory, Drug Radiation Research Department, NCRRT, Cairo, Egypt. It is worth mentioning that the 0.5 McFarland standard of all tested inoculums was set at 2–5 × 10^8^ CFU ml^−1^ (for bacteria) and 1–4 × 10^7^ CFU ml^−1^ (for yeast). The repression of the tested bacteria and yeast was determined by the zone of inhibition (ZOI) method after 24 h of incubation.^28^

Standard antibiotic discs (diameter of 6.0 mm), such as amoxicillin/clavulanic acid (AMC) and nystatin (NS), were used for comparison of the action of the examined samples.^29^

The minimum inhibitory concentration (MIC) was determined using Luria–Bertani (LB) broth with appropriate serial dilution.^30,31^ The microorganism and the nutrient served as a positive control, and the nutrient alone was used as a negative control. The Co_*x*_Ni_1−*x*_Fe_2_O_4_; *x* = 0.9/SiO_2_/TiO_2_ nanocomposite (starting with a concentration of 100 mg ml; 100 ppm) was tested. MIC values were calculated after 24 h of incubation at 37 °C.^32,33^ The tested bacterial inoculums were set at 3–4 × 10^8^ CFU ml^−1^, and at 2–4 × 10^7^ CFU ml^−1^ for the tested yeasts. MIC values were determined through enzyme-linked immunosorbent assay (ELISA) after fixing the absorption wavelength at 600 nm.^33,34^

#### 2.2.8. Antibiofilm activity of Co_*x*_Ni_1−*x*_Fe_2_O_4_; *x* = 0.9/SiO_2_/TiO_2_ nanocomposite

A semi-quantitative study of bacterial and yeast biofilm development was assessed according to the approach described by Christensen *et al.*^35^ Observations of the bacterial and yeast biofilm created throughout the interior walls of the test tubes were recorded, without and with the Co_*x*_Ni_1−*x*_Fe_2_O_4_/SiO_2_/TiO_2_; *x* = 0.9 nanocomposite. The antibiofilm action of the Co_*x*_Ni_1−*x*_Fe_2_O_4_/SiO_2_/TiO_2_; *x* = 0.9 nanocomposite at a concentration of 10.0 μg ml^−1^ was tested against pathogenic bacteria and *Candida* species and was compared with a control sample (a test tube without Co_*x*_Ni_1−*x*_Fe_2_O_4_/SiO_2_/TiO_2_; *x* = 0.9 nanocomposite).

In addition, nutrient broth (5.0 ml) was added to the test tubes after setting the 0.5 McFarland standard at 1–2 × 10^7^ CFU ml^−1^ (for the examined bacteria) and the tubes were then incubated for 24 h at 37 °C. The content of the control and treated tubes was discarded, and then all tubes were cleaned and rinsed with phosphate-buffered saline (PBS) with a pH of 7.5. Finally, all tubes were dried.^36,37^ The disciple bacterial and yeast films were fixed using sodium acetate (3%; 5 ml) for 10 min, and then tubes were rinsed with DI water. Bacterial and yeast biofilms were stained with crystal violet (CV; 0.1%) for 10 min and then soaked with DI water to remove excess CV.^38^ Subsequently, 4 ml of absolute ethanol was added to disintegrate the CV. The developed biofilms were recognized by the notable stained bands at the inner walls and the bottom of the test tubes.^39^ The bacterial and yeast biofilms were examined using a UV-vis spectrophotometer at 570 nm, and the biofilm repression percentage (%) was defined using eqn (1).^37,40^1
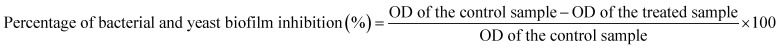


#### 2.2.9. Effect of UV irradiation

To define the impact of UV irradiation on the antimicrobial activity of Co_*x*_Ni_1−*x*_Fe_2_O_4_/SiO_2_/TiO_2_; *x* = 0.9 nanocomposite against the tested microbes, the percentage inhibition was determined using the optical density (OD) method.^41^ Three sensitive organisms were tested, including *E. coli* (Gram-negative bacterium), *S. aureus* (Gram-positive) and *C. albicans* (unicellular fungi). For each microbe, four test tubes were prepared. The first one was the control tube, which contained only tested microbes and was not irradiated with UV, the second control tube contained both microbes and the synthesized Co_*x*_Ni_1−*x*_Fe_2_O_4_; *x* = 0.9/SiO_2_/TiO_2_ nanocomposite and was not irradiated by UV, and the third tube contained only microbes and was irradiated by UV. Finally, the fourth tube contained both microbes and the tested Co_*x*_Ni_1−*x*_Fe_2_O_4_/SiO_2_/TiO_2_; *x* = 0.9 nanocomposite and was UV-irradiated.

All test tubes contained nutrient broth and a fixed count of microorganisms (0.5 McFarland; CFU ml^−1^). The UV-emitting tube (10 W low-pressure mercury lamp; 90% emittance at 254.0 nm) was located horizontally and fixed in the laminar flow, and the tested tubes were exposed to UV irradiation for 1 h at a distance of 2 feet (60.96 cm).

It is worth mentioning that the bacterial and yeast count was calculated every 10 min by UV-vis spectrophotometry at 600 nm (for bacteria) and 630 nm (for *Candida* species) for about 1 h and the inhibition percentage was measured according to eqn (1).

#### 2.2.10. Reaction mechanism using SEM and EDX analysis of treated bacterial cells

Bacterial cells obtained from the biofilm-creating experiment were rinsed with PBS and fitted with a 3.0% glutaraldehyde suspension. The dried bacterial samples were repeatedly cleaned by PBS and dried smoothly with different concentrations of ethanol (30.0%, 50.0%, 70.0%, 80.0%, 95.0%, and 100%) for 15 min at 28.0 ± 2.0 °C, which was a significant feature for dehydrating.^36^ Then, bacterial cells were set on an aluminum grip for SEM analysis.^36^ The morphological features of the control (non-treated bacterial cells) and cells treated with the fabricated Co_*x*_Ni_1−*x*_Fe_2_O_4_/SiO_2_/TiO_2_; *x* = 0.9 nanocomposite were examined through SEM and EDX investigations.

#### 2.2.11. Statistical analysis

Statistical analysis of our data was implemented by using the one-way analysis of variance (ANOVA) test (at *P* < 0.05) applying Duncan's multiple range considerations and the least significant difference report (LSD).^42^ The results and data obtained were analyzed by SPSS software (version 15).

## 3 Results and discussion

### 3.1. Characterization of the synthesized Co_*x*_Ni_1−*x*_Fe_2_O_4_/SiO_2_/TiO_2_; *x* = 0.9 nanocomposite

#### 3.1.1. High-resolution transmission electron microscopy

**Fig. 1:**
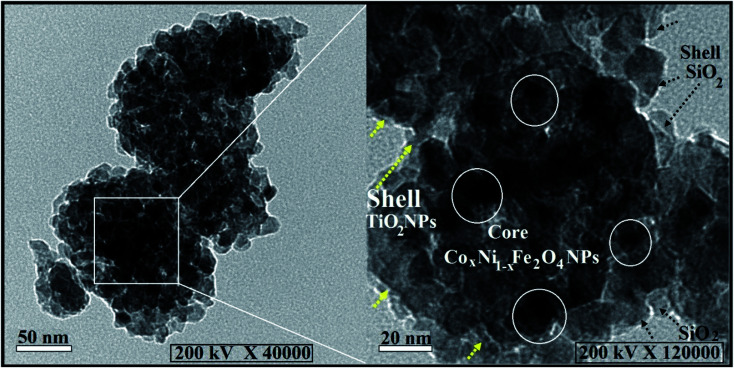
HRTEM analysis of the prepared Co*_x_*Ni_1−*x*_Fe_2_O_4_/SiO_2_/TiO_2_; *x* = 0.9 hybrid nanocomposite concentric structure, where yellow and black arrows display the two shell layers and the white circles represent the core Co_*x*_Ni_1−*x*_Fe_2_O_4_; *x* = 0.9 NPs.

HRTEM images of the concentric composition of the developed nanocomposite (Co_*x*_Ni_1−*x*_Fe_2_O_4_/SiO_2_/TiO_2_; *x* = 0.9) are presented in Fig. 1. The integrated composite has a semi-spherical construction with diameters varying from 90.56 nm to 155.50 nm, and an average diameter of 125.84 nm. It is worth noting that the compressed particles (white circles) are assigned to the magnetic core NPs (Co_*x*_Ni_1−*x*_Fe_2_O_4_; *x* = 0.9). Also, the hazy layers (black and yellow arrows) are attributed to the shell layers (SiO_2_ and TiO_2_ NPs), respectively. The construction of the composite and the arrangement of its layers were fully validated by SEM color mapping.

#### 3.1.2. Scanning electron microscopy and energy dispersive X-ray spectroscopic analysis of the synthesized nanocomposite

**Fig. 2:**
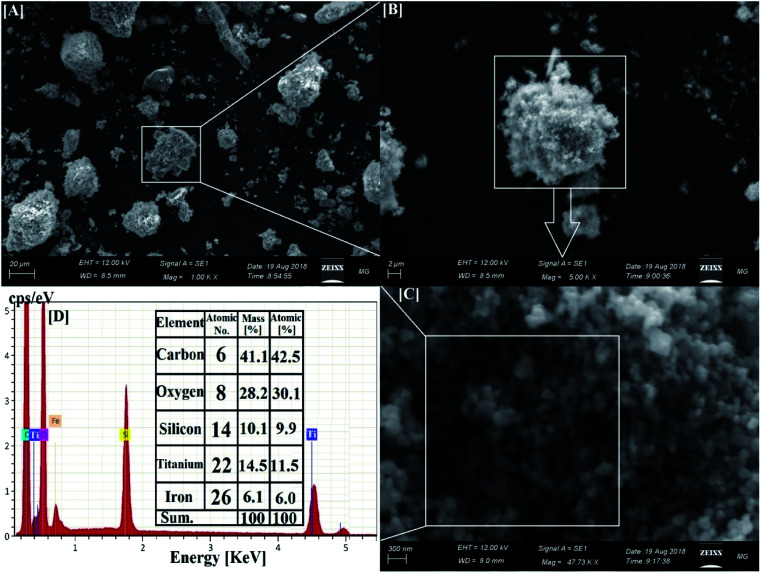
SEM and corresponding EDX elemental analysis of the synthesized Co*_x_*Ni_1−*x*_Fe_2_O_4_/SiO_2_/TiO_2_; *x* = 0.9 nanocomposite. [A–C] Different magnifications of the core–multi-shell structure. [D] The corresponding EDX elemental analysis of the synthesized nanocomposite.

SEM was used to examine the formation and morphology of the nanocomposite,^43^ while EDX examination was performed for elemental analysis and purity estimation.^44–46^ SEM images of the fabricated nanocomposite are presented in Fig. 2, where Co_*x*_Ni_1−*x*_Fe_2_O_4_; *x* = 0.9 NPs are located at the core, and the following two layers (of SiO_2_ and TiO_2_ NPs) are shells around this core, producing a core–multi-shell system.

Fig. 2 shows SEM and EDX analysis of the synthesized Co_*x*_Ni_1−*x*_Fe_2_O_4_/SiO_2_/TiO_2_; *x* = 0.9 nanocomposite revealing its particle dispersion with high purity through the appearance of C, O, Si, Ti and Fe atoms and the lack of any foreign elements (impurities). The recorded carbon atoms are attributed to the sample holder used in the SEM imaging.^47^ The corresponding EDX elemental analysis confirmed the presence of all the constituent atoms of the nanocomposite. The absence of Ni and Co atoms is attributed to their location at the deep core of the composite and their smaller ratios.

#### 3.1.3. Chemical mapping of the synthesized Co_*x*_Ni_1−*x*_Fe_2_O_4_/SiO_2_/TiO_2;_*x* = 0.9 nanocomposite

**Fig. 3:**
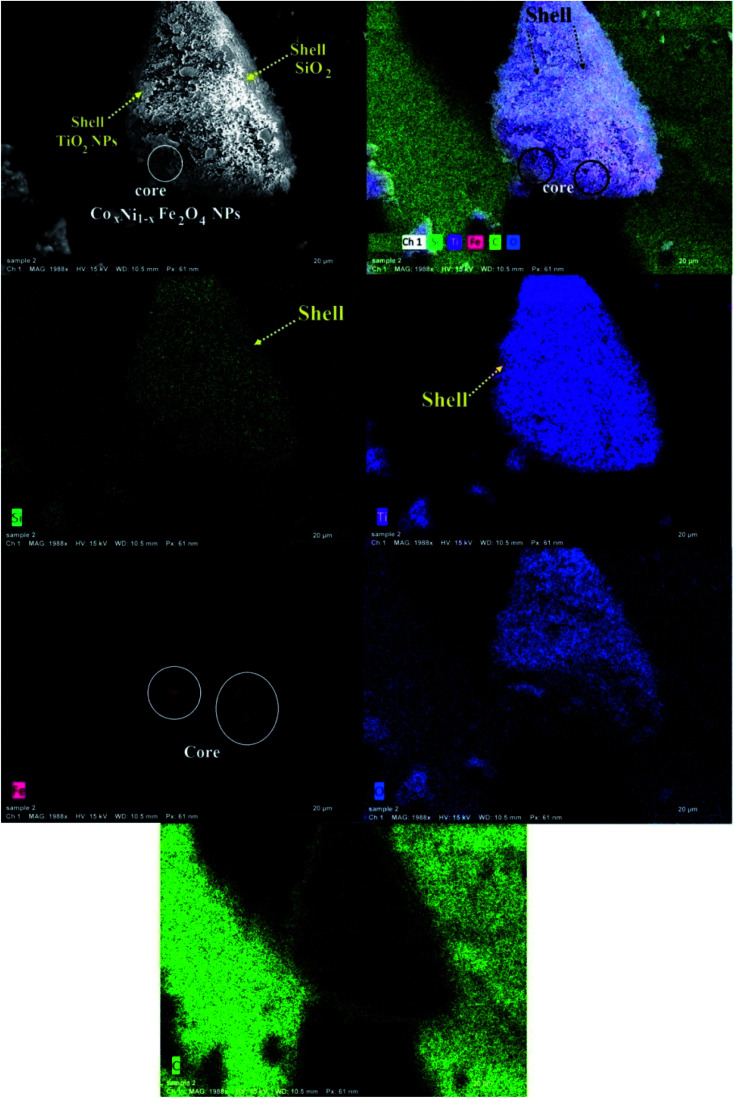
SEM-EDX elemental mapping of the synthesized Co*_x_*Ni_1−*x*_Fe_2_O_4_/SiO_2_/TiO_2_; *x* = 0.9 nanocomposite.

Elemental mapping of the integrated composite is shown in Fig. 3. The models were Si, Ti, Fe, O, and C. Fig. 3 verified the formation of the composite in terms of its components (Si, Ti, O, and Fe atoms). Interestingly, the images of elemental mapping confirmed the creation of a core–multi-shell system, revealed by the brightness and darkness of its layers. Co_*x*_Ni_1−*x*_Fe_2_O_4_; *x* = 0.9 NPs, which are located at the core, were relatively darker than both the SiO_2_ and TiO_2_ layers shielding that core.^48^ The brightest relative intensity of the Ti atoms showed that the TiO_2_ layer is the external layer and has the highest ratio (yellow arrow), followed by another relatively less bright layer attributed to Si atoms (yellow arrow). Finally, Fe atoms have a faint red color due to their location at the core with the smallest ratio (white circle; Fig. 3). Furthermore, both Co and Ni atoms (the principal metals in the ferrite) disappeared because they are located at the core.

This is one of the first times elemental mapping has been used as a tool to illustrate the development of a concentric structure, giving a promising explanation about the distribution and purity of the atoms and layers forming the nanocomposites.^49–51^

### 3.2. *In vitro* antimicrobial activity of the synthesized Co_*x*_Ni_1−*x*_Fe_2_O_4_/SiO_2_/TiO_2_; *x* = 0.9 nanocomposite

The disc agar spread method (which was performed as a screening step) revealed that the synthesized Co_*x*_Ni_1−*x*_Fe_2_O_4_/SiO_2_/TiO_2_; *x* = 0.9 nanocomposite displayed a relatively high antimicrobial activity against all examined bacteria and *Candida* species pathogens. Screening results confirmed that the Co_*x*_Ni_1−*x*_Fe_2_O_4_/SiO_2_/TiO_2_; *x* = 0.9 nanocomposite exhibited the highest antibacterial ability against *E. coli* (16.0 mm ZOI) and *S. aureus* (MRSA) (11.0 mm ZOI) as shown in Table 1.

**Table 1:** Antibacterial and antifungal activities of non-irradiated Co*_x_*Ni_1−*x*_Fe_2_O_4_/SiO_2_/TiO_2_; *x* = 0.9 nanocomposite, Co*_x_*Ni_1−*x*_Fe_2_O_4_; *x* = 0.9 NPs, SiO_2_ NPs, TiO_2_ NPs and DMSO, against multi-drug-resistant bacteria and pathogenic *Candida* species measured as ZOI (mm) and MIC (μg ml^−1^)^a^

Pathogenic microbes	ZOI of Co_*x*_Ni_1−*x*_Fe_2_O_4_/SiO_2_/TiO_2_; *x* = 0.9 NPs (mm)	MIC of Co_*x*_Ni_1−*x*_Fe_2_O_4_/SiO_2_/TiO_2_; *x* = 0.9 NPs (μg ml^−1^)	ZOI of Co_*x*_Ni_1−*x*_Fe_2_O_4_; *x* = 0.9 NPs; *x* = 0.9 (mm)	ZOI of SiO_2_ NPs (mm)	ZOI of TiO_2_NPs (mm)	ZOI of DMSO (mm)	AMC or NS
*Escherichia coli*	16.0^e^ ± 0.5000	3.12	10.0^e^ ± 0.1527	7.0^a^ ± 0.3214	7.0^a^ ± 0.2000	Nil	Nil
*Pseudomonas aeruginosa*	10.0^c^ ± 0.2886	12.5	9.0^d^ ± 0.2886	7.0^a^ ± 0.4618	8.0^b^ ± 0.3055	Nil	Nil
*Staphylococcus aureus*; MRSA	11.0^d^ ± 0.2645	6.25	8.0^c^ ± 0.4358	Nil	7.0^a^ ± 0.2886	Nil	Nil
*Bacillus subtilus*	10.0^c^ ± 0.5196	12.5	7.0^a^ ± 0.3214	Nil	Nil	Nil	Nil
*Proteus mirabilis*	10.0^c^ ± 0.2886	12.5	8.0^bc^ ± 0.2886	7.0^a^ ± 0.4163	7.0^a^ ± 0.4725	Nil	Nil
*Salmonella typhi*	9.0^b^ ± 0.2645	12.5	7.0^abc^ ± 0.5507	Nil	Nil	Nil	Nil
*Proteus vulgaris*	8.0^a^ ± 0.2645	25.0	8.0^a^ ± 0.2886	8.0^b^ ± 0.4509	8.0^b^ ± 0.3055	Nil	Nil
*Klebsiella pneumoniae*	9.0^b^ ± 0.0577	25.0	7.0 ^ab^ ± 0.4509	7.0^a^ ± 0.4932	8.0^b^ ± 0.2309	Nil	Nil
*Candida albicans*	10.0^c^ ± 0.4041	12.5	8.0^c^ ± 0.5196	8.0^b^ ± 0.4000	7.0^a^ ± 0.3214	Nil	Nil
*Candida tropicalis*	9.0^b^ ± 0.4000	25.0	7.0 ^ab^ ± 0.4509	7.0^a^ ± 0.3214	Nil	Nil	Nil
LSD	0.70000	—	1.33333	1.00000	0.76667	—	—

a Values are presented as mean ± SD (*n* = 3). ^a–e^Data within the groups were analyzed using one-way analysis of variance (ANOVA) followed by Duncan's multiple range test (DMRT). LSD, least significant difference. Nil, no ZOI measured. AMC, amoxicillin/clavulanic acid (standard antibacterial agent). NS, nystatin (standard antifungal agent).

It is of interest to note that the Co_*x*_Ni_1−*x*_Fe_2_O_4_/SiO_2_/TiO_2_; *x* = 0.9 nanocomposite has more powerful potential in terms of antimicrobial abilities than its separate layers (Co_*x*_Ni_1−*x*_Fe_2_O_4_; *x* = 0.9 NPs, SiO_2_, TiO_2_ NPs), DMSO and standard antimicrobial agents (AMC). It is also worth noting that the synthesized Co_*x*_Ni_1−*x*_Fe_2_O_4_/SiO_2_/TiO_2;_*x* = 0.9 nanocomposite was more active against Gram-negative bacteria than against Gram-positive species. The reasons for this effect may be due to the fact that the cell walls of Gram-negative species essentially consist of layers of peptidoglycan, lipopolysaccharide, and lipid, while the cell walls of Gram-positive species just have thick peptidoglycan structures.^52^

In addition, the produced Co_*x*_Ni_1−*x*_Fe_2_O_4_/SiO_2_/TiO_2_; *x* = 0.9 nanocomposite was very promising as an antifungal agent, conferring great antifungal efficiency towards *C. albicans* (10.0 mm ZOI), as presented in Table 1.

The MIC results for the Co_*x*_Ni_1−*x*_Fe_2_O_4_/SiO_2_/TiO_2_; *x* = 0.9 nanocomposite against all examined pathogenic bacteria and *Candida* species were in the range 12.5–3.125 μg ml^−1^, as shown in Table 1. The Co_*x*_Ni_1−*x*_Fe_2_O_4_/SiO_2_/TiO_2_; *x* = 0.9 nanocomposite possesses a promising MIC value of 3.125 μg ml^−1^ against *E. coli* and 6.25 μg ml^−1^ against *S. aureus*.

**Fig. 4:**
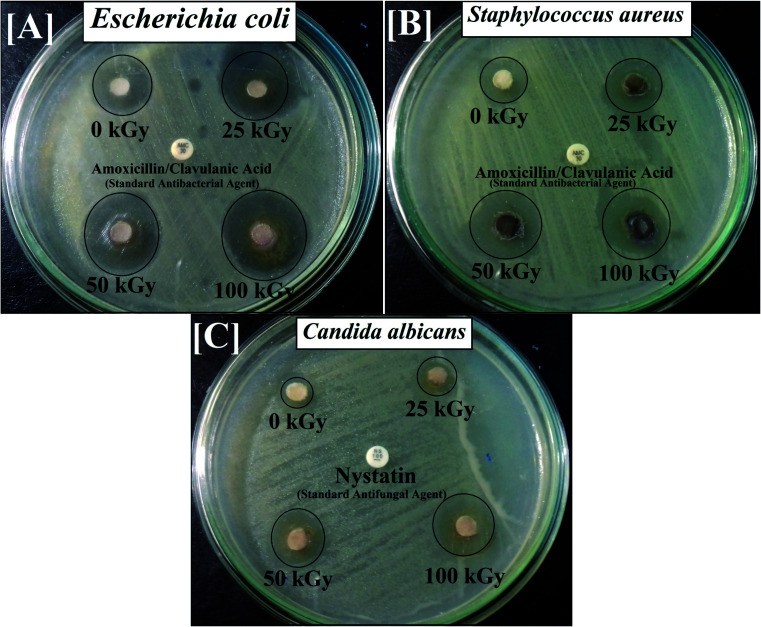
Antibacterial and antifungal activities of gamma-irradiated Co*_x_*Ni_1−*x*_Fe_2_O_4_/SiO_2_/TiO_2_; *x* = 0.9 nanocomposite against pathogenic microbes for [A] *E. coli*, [B] *Staphylococcus aureus* and [C] *C. albicans*, measured as ZOI (mm).

Subsequently, following gamma irradiation with doses of 25.0, 50.0 and 100.0 kGy, the antimicrobial activity of the synthesized Co_*x*_Ni_1−*x*_Fe_2_O_4_/SiO_2_/TiO_2_; *x* = 0.9 nanocomposite was evaluated. Gamma-irradiated Co_*x*_Ni_1−*x*_Fe_2_O_4_/SiO_2_/TiO_2_; *x* = 0.9 nanocomposite was more active against *E. coli* (30.0 mm ZOI; Fig. 4A), *S. aureus* (MRSA) (25.0 mm ZOI; Fig. 4B) and *C. albicans* (24.0 mm ZOI; Fig. 4C), as presented in Table 2.

**Table 2:** Antibacterial and antifungal activities of non-irradiated and gamma-irradiated Co_*x*_Ni_1−*x*_Fe_2_O_4_; *x* = 0.9/SiO_2_/TiO_2_ nanocomposite against pathogenic microbes measured as ZOI (mm) and MIC (μg ml^−1^)^a^

Pathogenic microbes	ZOI of non-irradiated Co_*x*_Ni_1−*x*_Fe_2_O_4_/SiO_2_/TiO_2_; *x* = 0.9 NPs (mm)	ZOI of irradiated Co_*x*_Ni_1−*x*_Fe_2_O_4_/SiO_2_/TiO_2_; *x* = 0.9 NPs (25.0 kGy) (mm)	ZOI of irradiated Co_*x*_Ni_1−*x*_Fe_2_O_4_/SiO_2_/TiO_2_; *x* = 0.9 NPs (50.0 kGy) (mm)	ZOI of irradiated Co_*x*_Ni_1−*x*_Fe_2_O_4_/SiO_2_/TiO_2_; *x* = 0.9 NPs (100.0 kGy) (mm)	MIC of irradiated Co_*x*_Ni_1−*x*_Fe_2_O_4_/SiO_2_/TiO_2_; *x* = 0.9 NPs (100.0 kGy) (μg ml^−1^)	AMC or NS
*Escherichia coli*	17.0^b^ ± 0.3214	25.0^f^ ± 0.4509	27.0^h^ ± 0.2645	30.0^h^ ± 0.4163	0.024	Nil
*Pseudomonas aeruginosa*	11.0^a^ ± 0.2645	18.0^d^ ± 0.4509	19.0^e^ ± 0.2516	20.0^e^ ± 0.2516	0.390	Nil
*Staphylococcus aureus*; MRSA	12.0^a^ ± 0.1808	19.0.0^e^ ± 0.5507	22.0^g^ ± 0.2081	25.0^g^ ± 0.2645	0.097	Nil
*Bacillus subtilus*	10.0^a^ ± 0.4725	12.0.0^b^ ± 0.2645	14.0^d^ ± 0.3214	16.0^d^ ± 0.4582	1.562	Nil
*Proteus mirabilis*	11.0^a^ ± 0.2000	13.0^a^ ± 0.2516	13.0^c^ ± 0.2081	15.0^c^ ± 0.5000	3.125	Nil
*Salmonella typhi*	10.0^a^ ± 0.4509	11.0^a^ ± 0.2516	11.0^a^ ± 0.1527	12.0^a^ ± 0.2645	6.250	Nil
*Proteus vulgaris*	10.0^a^ ± 0.4509	12.0^b^ ± 0.2886	14.0^d^ ± 0.4000	16.0^d^ ± 0.3464	3.125	Nil
*Klebsiella pneumoniae*	11.0^a^ ± 0.2516	11.0^a^ ± 0.3055	12.0^b^ ± 0.3214	13.0^b^ ± 0.2516	6.250	Nil
*Candida albicans*	10.0^a^ ± 0.4509	18.0^d^ ± 0.4000	20.0^f^ ± 0.4041	24.0^f^ ± 0.2886	0.195	Nil
*Candida tropicalis*	10.0^a^ ± 0.1527	12.0^b^ ± 0.3214	14.0^d^ ± 0.2886	15.0^c^ ± 0.2516	6.250	Nil
LSD	6.06666	0.83333	0.90000	0.90000	—	—

a Values are presented as mean ± SD (*n* = 3). ^a–e^Data within the groups were analyzed using one-way analysis of variance (ANOVA) followed by Duncan's multiple range test (DMRT). LSD, least significant difference. Nil, no ZOI measured. AMC, amoxicillin/clavulanic acid (standard antibacterial agent). NS, nystatin (standard antifungal agent).

Interestingly, the MIC values decreased with increasing dose of gamma rays and a superior MIC result was recorded at 0.024 μg ml^−1^ against *E. coli* for the Co_*x*_Ni_1−*x*_Fe_2_O_4_/SiO_2_/TiO_2;_*x* = 0.9 nanocomposite irradiated by 100.0 kGy.

**Fig. 5:**
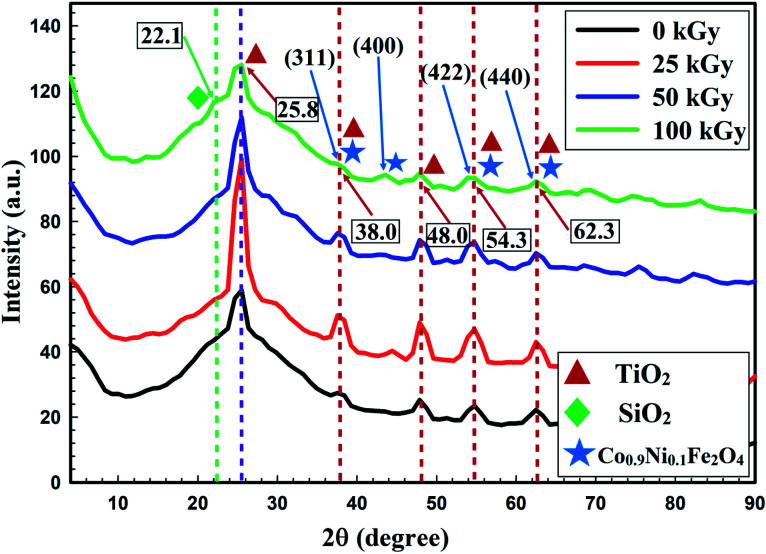
XRD pattern showing the effect of gamma irradiation, with doses of 25, 50 and 100 kGy, on the crystal size of the prepared Co*_x_*Ni_1−*x*_Fe_2_O_4_/SiO_2_/TiO_2_; *x* = 0.9 nanocomposite, compared with the non-irradiated composite.

The enhanced activity of the prepared nanocomposite against all tested microorganisms after gamma irradiation was due to the reduction in crystallite size (from 44.2 nm to 17.4 nm) after 100 kGy irradiation.^53,54^ The XRD patterns of non-irradiated and gamma-irradiated Co_*x*_Ni_1−*x*_Fe_2_O_4_/SiO_2_/TiO_2_; *x* = 0.9 nanocomposite with different doses (25 kGy, 50 kGy and 100 kGy) are shown in Fig. 5.

The XRD patterns presented in Fig. 5 show the crystallinity of the non-irradiated and gamma-irradiated Co_*x*_Ni_1−*x*_Fe_2_O_4_/SiO_2_/TiO_2_; *x* = 0.9 nanocomposite. Characteristic peaks of TiO_2_ NPs, SiO_2_ NPs and Co_*x*_Ni_1−*x*_Fe_2_O_4_; *x* = 0.9 NPs were clearly recorded. Many sharp, strong and intense peaks were observed at 2*θ* values of 25.8° (reflection 101), 38.0° (reflection 112), 48.0° (reflection 200), 54.3° (reflection 105), and 60.3° (reflection 213), while the principal peak was observed at 2*θ* = 25.8°. Recorded peaks matched those of TiO_2_ NPs (JCPDS 04-0477).^24^

An amorphous extended peak was recorded at 2*θ* = 22.1°, which is associated with the interatomic lengths in SiO_2_ NPs.^55–58^ Finally, sharp and intense peaks were observed at 2*θ* = 38.0° (reflection 311), 45.2° (reflection 400), 54.3° (reflection 422), and 62.3° (reflection 440), while a promising peak appeared at 38.0°, registering the appearance of both Ni Fe_2_O_4_ (JCPDS 10-325) and Co Fe_2_O_4_ (JCPDS 1-1121), indicating the development of Co_*x*_Ni_1−*x*_Fe_2_O_4_; *x* = 0.9 NPs.^59^

The average crystallite size was calculated using the Debye–Scherrer equation as:
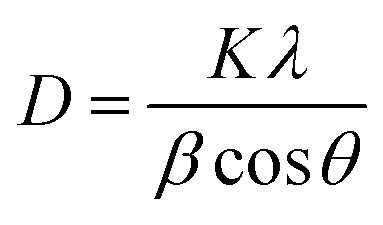
where *K* = 0.9 and is known as the shape factor, *λ* is the wavelength of the X-ray (1.54060 Å for CuKα), *β* is the full width at half maximum (FWHM) and *θ* is the diffraction angle.

From Fig. 5, it can be seen that crystallite size was reduced by increasing the gamma radiation dose from 25 to 100 kGy. This influence may be due to the movement of gamma rays inside the materials, transporting its power in a compact time interval within flexible contact with particles,^53^ which leads to modification in the irradiated material by forcing the atoms from their initial conditions, and dividing particles.^54^

Additionally, with increase in irradiation dose the samples showed a tendency to become amorphous (Fig. 5) and this is because the crystallite size is independent of the crystallinity of the sample. Crystallization of a material means that there are long arrangements of atoms. This can be indicated by the intensity of the XRD lines. For XRD, the smallest crystallite size caused obvious broadening of the XRD peaks, but crystallite size gives information about the formed sample.^60^ A perfect crystal would extend infinitely in all directions; hence, no crystals are perfect because of their finite crystal size. This deviation from perfect crystallinity leads to broadening of the diffraction peaks.^61^ The two principal properties obtained from peak width analysis are the crystallite size and lattice strain.

Crystallite size is a measure of the size of coherently diffracting domains. The crystallite size of the particles is not usually the same as the particle size due to the production of polycrystalline aggregates.^62^ Lattice strain is a measure of the distribution of lattice constants resulting from crystal imperfections, such as lattice dislocations. Other sources of strain include the grain boundary triple junction, contact or sinter stresses, stacking faults, and coherency stresses.^63^ Crystallite size and lattice strain affect the Bragg peak in different ways. Both these effects extend the peak width and intensity and shift the 2*θ* peak position accordingly.

Interestingly, there is a relationship between the physical properties of the fabricated Co_*x*_Ni_1−*x*_Fe_2_O_4_/SiO_2_/TiO_2_; *x* = 0.9 nanocomposite and its recorded antimicrobial abilities, with surface area playing a significant role in the antimicrobial action of the synthesized Co_*x*_Ni_1−*x*_Fe_2_O_4_/SiO_2_/TiO_2_; *x* = 0.9 nanocomposite against all tested pathogenic microbes.

The calculated surface area was 46.13 m^2^ g^−1^, with a broad spread of pore sizes of TiO_2_ NPs, with an average value of 3.71 nm and an average pore volume of about 0.19 cm^3^ g^−1^, as previously calculated in our recent paper,^24^ implying the appearance of two kinds of pores in the external layer of the composite TiO_2_ NPs: mesopores and macropores.^64^

The high surface area and broad pore size of the produced nanocomposite increased the contact area to the microbial cell and hence the antimicrobial activity of the Co*_x_*Ni_1−*x*_Fe_2_O_4_/SiO_2_/TiO_2_; *x* = 0.9 nanocomposite was increased.

The gamma-irradiated Co_*x*_Ni_1−*x*_Fe_2_O_4_/SiO_2_/TiO_2_; *x* = 0.9 nanocomposite had a more stable profile, with relatively lower crystal size (19.6 nm, 19.3 nm and 17.4 nm after gamma irradiation with 25 kGy, 50 kGy and 100 kGy, respectively; Fig. 5) compared to the non-irradiated composite. These physical properties were critical in improving its antimicrobial efficacy even at a low concentration (0.024 μg ml^−1^) against all examined pathogenic bacteria and *Candida* species.

### 3.3. Antimicrobial potential of UV-irradiated Co_*x*_Ni_1−*x*_Fe_2_O_4_/SiO_2_/TiO_2_; *x* = 0.9 nanocomposite in liquid media

**Fig. 6:**
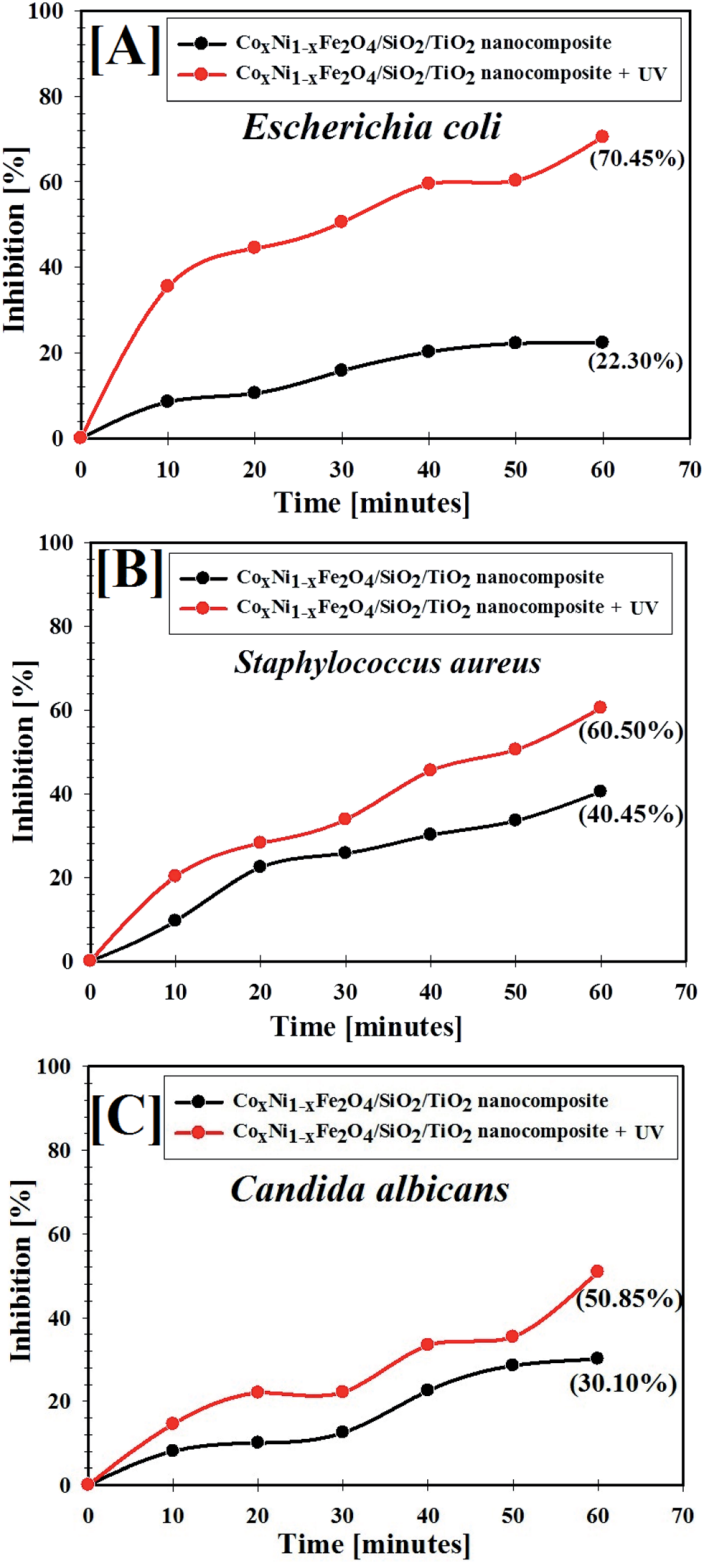
Antimicrobial abilities of UV-irradiated Co*_x_*Ni_1−*x*_Fe_2_O_4_/SiO_2_/TiO_2_; *x* = 0.9 nanocomposite against different pathogens: [A] *E. coli*, [B] *S. aureus* and [C] *C. albicans*.

A comparative study of the inhibition percentage of *E. coli*, *S. aureus*, and *C. albicans* by non-irradiated Co_*x*_Ni_1−*x*_Fe_2_O_4_/SiO_2_/TiO_2_; *x* = 0.9 nanocomposite and UV-irradiated Co_*x*_Ni_1−*x*_Fe_2_O_4_/SiO_2_/TiO_2_; *x* = 0.9 nanocomposite was performed and is presented in Fig. 6.

As shown in Fig. 6A–C, the inhibition percentage of the tested pathogens increased with time upon treatment with the Co_*x*_Ni_1−*x*_Fe_2_O_4_/SiO_2_/TiO_2_; *x* = 0.9 nanocomposite, meaning that the Co_*x*_Ni_1−*x*_Fe_2_O_4_/SiO_2_/TiO_2_; *x* = 0.9 nanocomposite showed antimicrobial activities against colonies of *E. coli*, *S. aureus* and *C. albicans*. Interestingly, the UV-irradiated Co_*x*_Ni_1−*x*_Fe_2_O_4_/SiO_2_/TiO_2_; *x* = 0.9 nanocomposite exhibited superior antimicrobial activities compared with the non-irradiated nanocomposite, as shown in Fig. 6. The maximum inhibition percentage of non-irradiated Co_*x*_Ni_1−*x*_Fe_2_O_4_/SiO_2_/TiO_2_; *x* = 0.9 nanocomposite and UV-irradiated Co_*x*_Ni_1−*x*_Fe_2_O_4_/SiO_2_/TiO_2_; *x* = 0.9 nanocomposite against *E. coli* was 22.30% and 70.45%, respectively, and the inhibition was 40.45% and 60.50% for *S. aureus* and 30.10% and 50.85% for *C. albicans*, after 60 min (experiment time).

It was found that the Co_*x*_Ni_1−*x*_Fe_2_O_4_/SiO_2_/TiO_2_; *x* = 0.9 nanocomposite was more active under UV irradiation, confirming the presence of photogenerated reactive oxygen species (ROS) that can decompose bacterial cells. Here, the Co_*x*_Ni_1−*x*_Fe_2_O_4_/SiO_2_/TiO_2_; *x* = 0.9 nanocomposite was shown to have good antimicrobial abilities attributed to its high UV absorption intensity. Hydroxyl (OH) radicals can also be generated by irradiating Co_*x*_Ni_1−*x*_Fe_2_O_4_/SiO_2_/TiO_2_; *x* = 0.9 nanocomposite with ultraviolet light, due to electron transfer between microbial cells and the nanocomposite. The OH radicals can destroy bacterial cells causing a reduction in cell coenzyme content.^65^

In addition, it has been reported that metal oxides (MO) display a positive charge in slightly acidic medium, while microbes possess a negative charge. This creates an electromagnetic affinity between microbes and the MO, leading to oxidization of microbial cells and their subsequent destruction.^66^ Additionally, nanomaterials can destroy cellular proteins and DNA by adhering to electron-donating structures, such as carbohydrates, thiols, indoles, amides, and hydroxyls. In addition, they can create holes in the bacterial cell walls, making them outwardly permeable and finally resulting in cell loss.^67^ We have previously reported that our composite possessed negative charge, but the media of the microbes is slightly acidic (pH = 6.0), which can change the surface charge of the composite to positive, and this is in a good agreement with our results (see Fig. 14B later).

### 3.4. Antibiofilm activity of Co_*x*_Ni_1−*x*_Fe_2_O_4_/SiO_2_/TiO_2_; *x* = 0.9 nanocomposite gamma-irradiated with 100 kGy

Biofilm creation has been recognized in several exopolysaccharide-forming microbes.^36,50^ Biofilm production by common pathogenic bacteria and yeast microorganisms in the absence and presence of gamma-irradiated (with 100 kGy) Co_*x*_Ni_1−*x*_Fe_2_O_4_/SiO_2_/TiO_2_; *x* = 0.9 nanocomposite was assessed using a test tube method.^68^

**Fig. 7:**
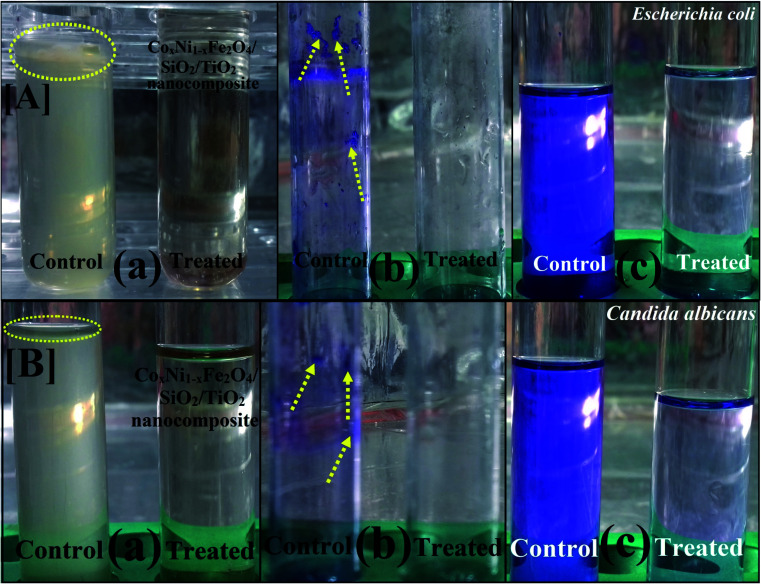
Antibiofilm activity of 100 kGy gamma-irradiated Co*_x_*Ni_1−*x*_Fe_2_O_4_/SiO_2_/TiO_2_; *x* = 0.9 nanocomposite using the test tube method against [A] *E. coli* and [B] *C. albicans*. The steps were reported as follows. (a) Growth of the bacterial and yeast cells and biofilm formation (rings) without treatment with the synthesized Co*_x_*Ni_1−*x*_Fe_2_O_4_/SiO_2_/TiO_2_; *x* = 0.9 nanocomposite and the inhibition of bacterial and yeast growth after treatment with Co*_x_*Ni_1−*x*_Fe_2_O_4_/SiO_2_/TiO_2_; *x* = 0.9 nanocomposite. (b) Staining of the adherent bacterial and yeast cells with crystal violet. (c) Removing and dissolving the adherent bacterial and yeast cells by ethanol for determination of semi-quantitative biofilm inhibition (%) (as shown in Table 3).

Fig. 7A shows the antibiofilm activity of 100 kGy gamma-irradiated Co_*x*_Ni_1−*x*_Fe_2_O_4_/SiO_2_/TiO_2_; *x* = 0.9 nanocomposite against *E. coli* bacteria (as a model for susceptible bacteria).

*E. coli* inoculated in the absence of gamma-irradiated Co_*x*_Ni_1−*x*_Fe_2_O_4_/SiO_2_/TiO_2_; *x* = 0.9 nanocomposite exhibited a thick whitish-yellow mat throughout the air–liquid interface. This mat was totally adhered to the wall of the test tubes and appeared as a blue ring after CV staining. A blue suspension was developed after dissolving the CV-stained ring with absolute ethanol, as displayed in Fig. 7A.

On the other hand, *E. coli*-inoculated test tubes that were treated with gamma-irradiated Co_*x*_Ni_1−*x*_Fe_2_O_4_/SiO_2_/TiO_2_; *x* = 0.9 nanocomposite (10.0 μg ml^−1^) exhibited a suppressed effect where the development of bacterial rings was limited. In addition, the blue color representing CV-stained adherent bacterial cells was light and, after CV dissolution by ethanol, no blue color was observed, as shown in Fig. 7A. A similar effect was recorded for the biofilm repression of *C. albicans* (as an example of a sensitive yeast), as presented in Fig. 7B.

To determine the repression percentage (%) of bacterial and yeast biofilm, a UV-vis spectrophotometer was used (at 570.0 nm). The optical density was determined after separating CV-stained bacterial and yeast biofilms with ethanol. Table 3 shows the inhibition percentage of the biofilms formed by the tested bacteria and yeast strains. The highest percentage inhibition was observed against *E. coli* (92.82%; Fig. 7A and Table 3) followed by *C. albicans* (92.29%; Fig. 7B and Table 3) and *S. aureus* (90.69%; Table 3) after treatment with 10.0 μg ml^−1^ of gamma-irradiated Co_*x*_Ni_1−*x*_Fe_2_O_4_/SiO_2_/TiO_2_; *x* = 0.9 nanocomposite.

**Table 3:** Semi-quantitative inhibition (%) of the biofilm formation for non-treated and treated bacterial and yeast pathogens with 100 kGy gamma-irradiated Co*_x_*Ni_1−*x*_Fe_2_O_4_/SiO_2_/TiO_2_; *x* = 0.9 nanocomposite^a^

Bacterial and yeast strains	OD of crystal violet stain at 570.0 nm (control)	OD of crystal violet stain at 570.0 nm (treated with 10.0 μg ml^−1^ Co_*x*_Ni_1−*x*_Fe_2_O_4_/SiO_2_/TiO_2_ nanocomposite)	Inhibition (%)
*Escherichia coli*	0.992^j^ ± 0.0017	0.071^d^ ± 0.0023	92.82%
*Pseudomonas aeruginosa*	0.598^e^ ± 0.0030	0.089^d^ ± 0.0020	85.11%
*Staphylococcus aureus* (MRSA)	0.645^f^ ± 0.0026	0.060^c^ ± 0.0026	90.69%
*Bacillus subtilus*	0.574^d^ ± 0.0023	0.098^f^ ± 0.0010	82.92%
*Proteus mirabilis*	0.411^a^ ± 0.0045	0.088^e^ ± 0.0047	78.58%
*Salmonella typhi*	0.897^i^ ± 0.0005	0.499^b^ ± 0.0001	44.37%
*Proteus vulgaris*	0.708^h^ ± 0.0010	0.166^h^ ± 0.0020	76.55%
*Klebsiella pneumoniae*	0.666^g^ ± 0.0030	0.313^i^ ± 0.0015	53.00%
*Candida albicans*	0.519^c^ ± 0.0026	0.040^a^ ± 0.0025	92.29%
*Candida tropicalis*	0.501^b^ ± 0.0026	0.111^g^ ± 0.0040	77.84%
LSD	0.01700	0.00767	—

a Values are presented as mean ± SD (*n* = 3). ^a–e^Data within the groups were analyzed using one-way analysis of variance (ANOVA) followed by Duncan's multiple range test (DMRT). LSD, least significant difference.

The synthesized 100 kGy gamma-irradiated Co_*x*_Ni_1−*x*_Fe_2_O_4_/SiO_2_/TiO_2_; *x* = 0.9 nanocomposite was used to restrain the development of biofilm at its adhesion step (identified as the primary step).^69^

The variance in the percentage inhibition may be attributed to various circumstances, such as antimicrobial action, biosorption (because of the large surface area of the synthesized nanocomposite), physical characteristics (particle size of the nanocomposite), invasion capabilities and different chemical features controlling the interaction of the nanocomposite and the biofilms.^68,70^ It was also found that the 100 kGy gamma-irradiated Co_*x*_Ni_1−*x*_Fe_2_O_4_/SiO_2_/TiO_2_; *x* = 0.9 nanocomposite inhibited *E. coli* by greater than 98% at 0.024 μg ml^−1^ MIC (Table 2). When the exopolysaccharide assembly is restricted (the principal molecules for biofilm development), *E. coli* cannot make a biofilm.^36,68^

**Fig. 8:**
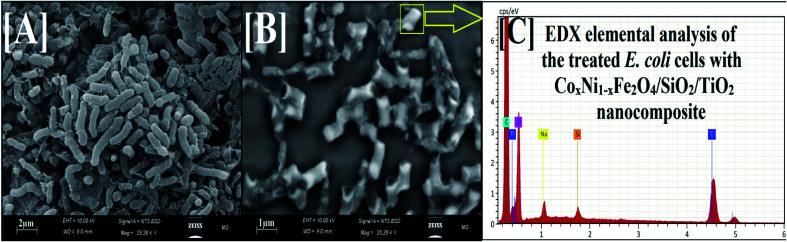
SEM and corresponding EDX elemental analysis of *E. coli*. [A] Normal bacterial cell without treatment with 100 kGy gamma-irradiated Co*_x_*Ni_1−*x*_Fe_2_O_4_/SiO_2_/TiO_2_; *x* = 0.9 nanocomposite. [B] The depressed and deformed bacterial cell after treatment with 100 kGy gamma-irradiated Co_*x*_Ni_1−*x*_Fe_2_O_4_; *x* = 0.9/SiO_2_/TiO_2_ nanocomposite (yellow square represents complete lysis of *E. coli* cells). [C] The corresponding EDX elemental analysis of the treated *E. coli* cell, confirming the cellular internalization of the 100 kGy gamma-irradiated Co_*x*_Ni_1−*x*_Fe_2_O_4_; *x* = 0.9/SiO_2_/TiO_2_ nanocomposite.

To further explain the antibiofilm abilities of the 100 kGy gamma-irradiated Co_*x*_Ni_1−*x*_Fe_2_O_4_/SiO_2_/TiO_2_; *x* = 0.9 nanocomposite, we propose a mechanism of action against biofilm production by *E. coli* from SEM and EDX studies.^37,71^ The SEM images show the bacterial cell morphologies before and after treatment with 100 kGy gamma irradiation of the prepared nanocomposite. In the control (non-treated bacterial cells), bacterial cultures were normally developed and had definite normal cellular morphology with a normal cell exterior and compressed biofilm production, as displayed in Fig. 8A.

After treatment, morphological modifications were recognized on *E. coli* cells (Fig. 8). There was an obvious change at the surface, followed by a deformation and decrease in the viable count of *E. coli* cells. Moreover, biofilm formation was arrested. SEM investigation also showed shrinkage of the bacterial cell walls^71^ (Fig. 8B), while the EDX elemental study showed the appearance of Ti and Si atoms (atoms of the outer shell of the prepared nanocomposite) at the shrinkage area and the external surface of the tested *E. coli* cells, confirming the reason for the recorded effect, namely nanocomposite treatment (Fig. 8C).

One probable cause for the reported activity against *E. coli* could be due to the large surface area (46.13 m^2^ g^−1^) enabling better static communication between the negatively charged cell walls of the examined bacteria,^72,73^ as exhibited in Fig. 8B.

This conclusion is in a reasonable agreement with several published reports confirming the interaction between MO NPs and pathogens through electrostatic power, leading to bacterial membrane separation.^72,74,75^ Recent research affirms that MO NPs increase the oxidative pressure against pathogens^76^ and rapidly destroy their cell membranes because of the high level of ROS generated. Additionally, detailed reaction mechanisms for MO NPs against pathogenic bacteria and yeast cells have been described in our previous studies.^37,77^

Herein, the prepared nanocomposite interacted externally with *E. coli* cells by means of electrostatic affinity and reduced the bacterial cell counts through membrane leakage.^74^ The proposed reaction mechanism begins with adhesion of the nanocomposite onto the surface of *E. coli*. After that, Ti^2+^ and Si^2+^ ions (from the outer shell) enter the tested bacterial cell and destroy its biological molecules, such as mitochondria and DNA. Then, cellular toxicity due to oxidative stress through the production of ROS develops. The nanocomposite can certainly block the signal transduction cycle of the examined bacterial cells.^78^

### 3.5. Photocatalytic activity of Co_*x*_Ni_1−*x*_Fe_2_O_4_/SiO_2_/TiO_2_; *x* = 0.9 nanocomposite

The photocatalytic activity of the synthesized Co_*x*_Ni_1−*x*_Fe_2_O_4_/SiO_2_/TiO_2_; *x* = 0.9 nanocomposite was determined using photocatalytic degradation of pyridine solution (Py) under visible light illumination.

**Fig. 9:**
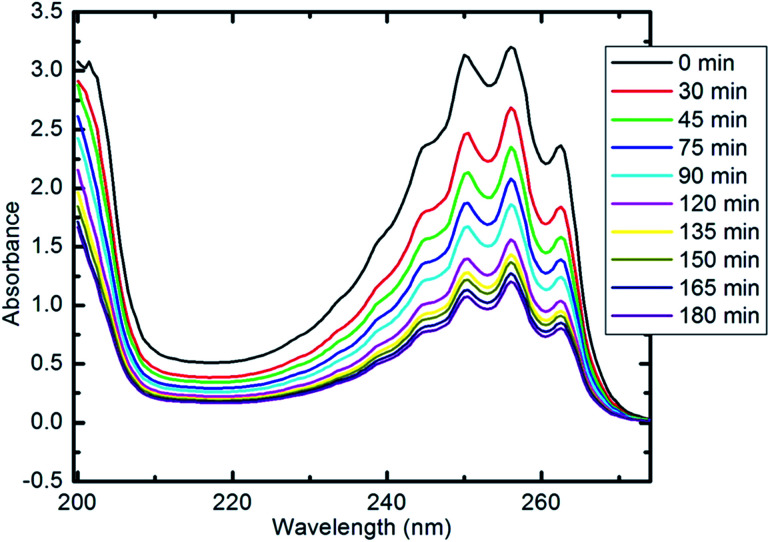
UV-visible spectra of pyridine showing its degradation with time (10 mg of nanocomposite, 50 ml Py solution (100 ppm), 25 °C and pH 7).

Fig. 9 illustrates the decline in absorption with irradiation time for the photocatalytic degradation of pyridine solution (50 ml, 100 mg l^−1^) using 10 mg of the prepared nanocomposite. With increasing irradiation period, the strong absorption bands of pyridine recorded at 256 nm (indicating the maximum wavelength, *λ*_max_, for the pyridine) are continuously reduced. The absorbance of the pyridine solution was reduced by 85% after 100 min of visible light irradiation at the specified experimental conditions.

#### 3.5.1. Effect of the initial concentration of pyridine on degradation efficiency

**Fig. 10:**
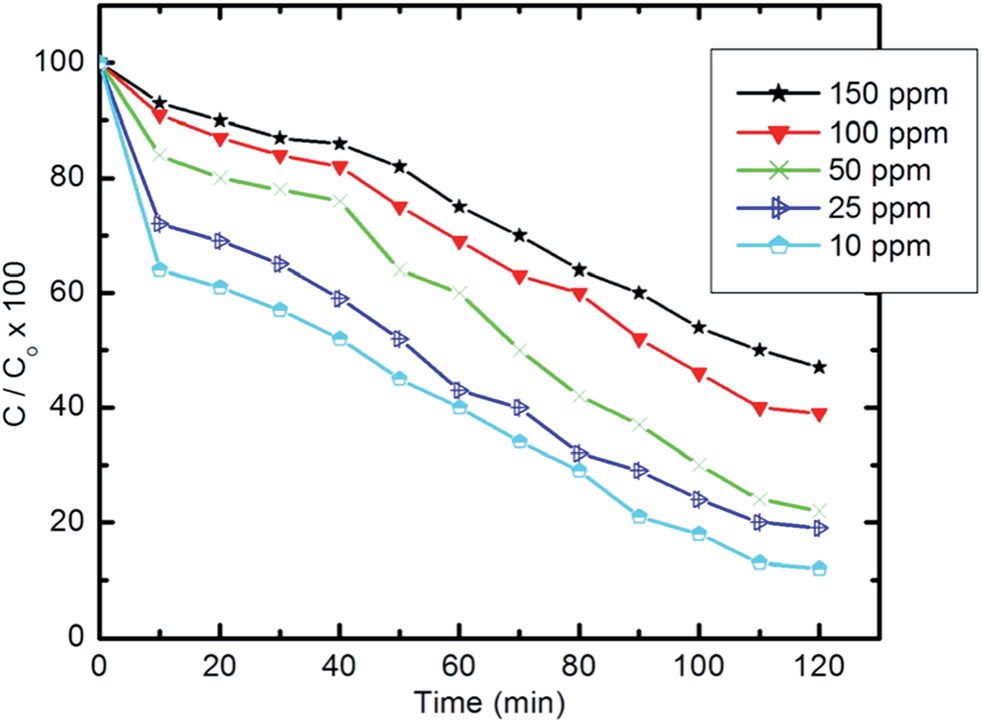
Effect of initial concentration of pyridine solution on the degradation efficiency (10 mg of composite, 50 ml Py solution, 25 °C and pH 7).

The degradation efficiency of the prepared nanocomposite against pyridine at different initial concentrations (10–150 ppm) is shown in Fig. 10. The results show that the degradation efficiency is inversely proportional to the concentration of pyridine, which can be effectively removed in the presence of the prepared nanocomposite under visible light irradiation even at high initial concentrations.

Table 4 gives the percentage degradation of pyridine at a range of high initial concentrations (300–1000 ppm) and also indicates the effectiveness of pyridine degradation over this range. Upon increasing the initial concentration from 300 to 1000 ppm, a minor decrease in degradation efficiency was observed. The measured degradation efficiencies exceeded 86%. The pyridine concentration removed from wastewater increases upon increasing the initial concentration without affecting the degradation efficiency.

**Table 4:** Removal of pyridine solution at high initial concentrations

Concentration (μg ml^−1^)	300	500	800	1000
Degradation percentage (%)	88.2	87.4	87.1	86.3

The degradation rate of pyridine can be calculated using the following equation:

2 ln *C*_*t*_/*C*_0_ = −*Kt*

where *C*_*t*_ and *C*_0_ are the remaining and initial concentrations of pyridine, respectively, *t* is the irradiation time and *K* represents the degradation rate constant.

**Fig. 11:**
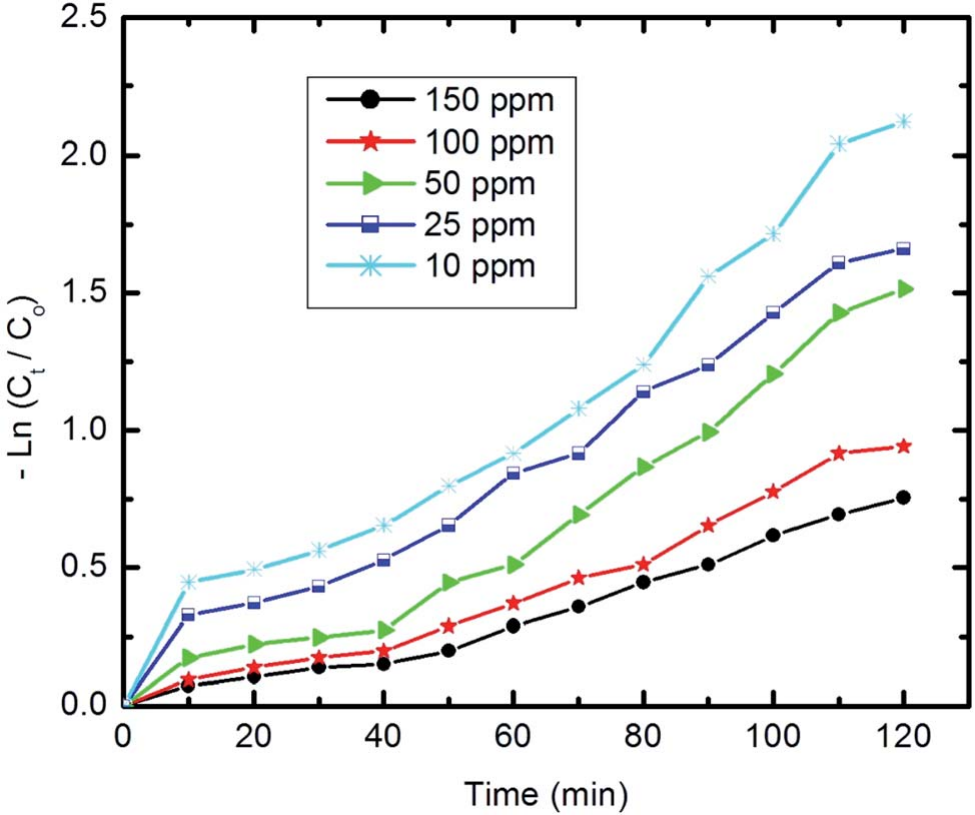
The first-order kinetics of pyridine degradation (10 mg of composite, 50 ml Py solution, 25 °C and pH 7).

**Fig. 12:**
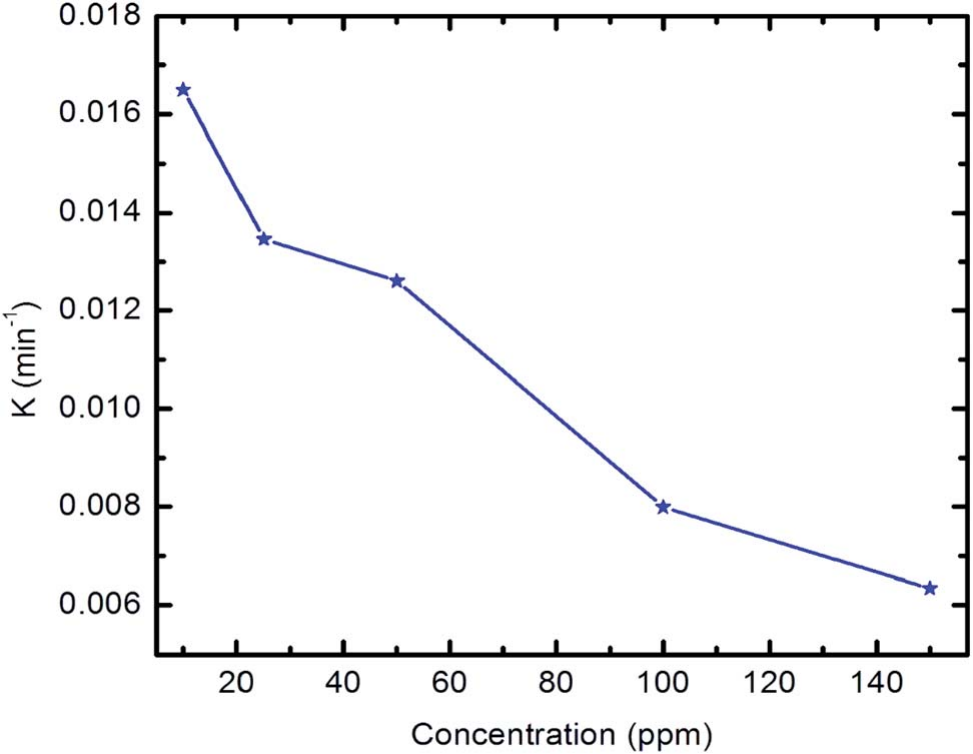
Apparent first-order rate constants *vs.* initial concentration of pyridine.

Fig. 11 shows the relationship of −ln *C*_*t*_/*C*_0_*vs.* time. The results show that the kinetics of the degradation reaction follow the laws of a first-order rate (*R*^2^ > 98) at initial concentrations. Moreover, as revealed by Fig. 12, there is an inverse relationship between the apparent first-order rate constants and initial pyridine concentration, which indicates the non-elementary nature of the photocatalytic reaction. This recorded dependence of the reaction rate constants on initial concentration is in a good agreement with the literature.^79,80^

#### 3.5.2. Effect of the dose of photocatalyst on degradation efficiency

**Fig. 13:**
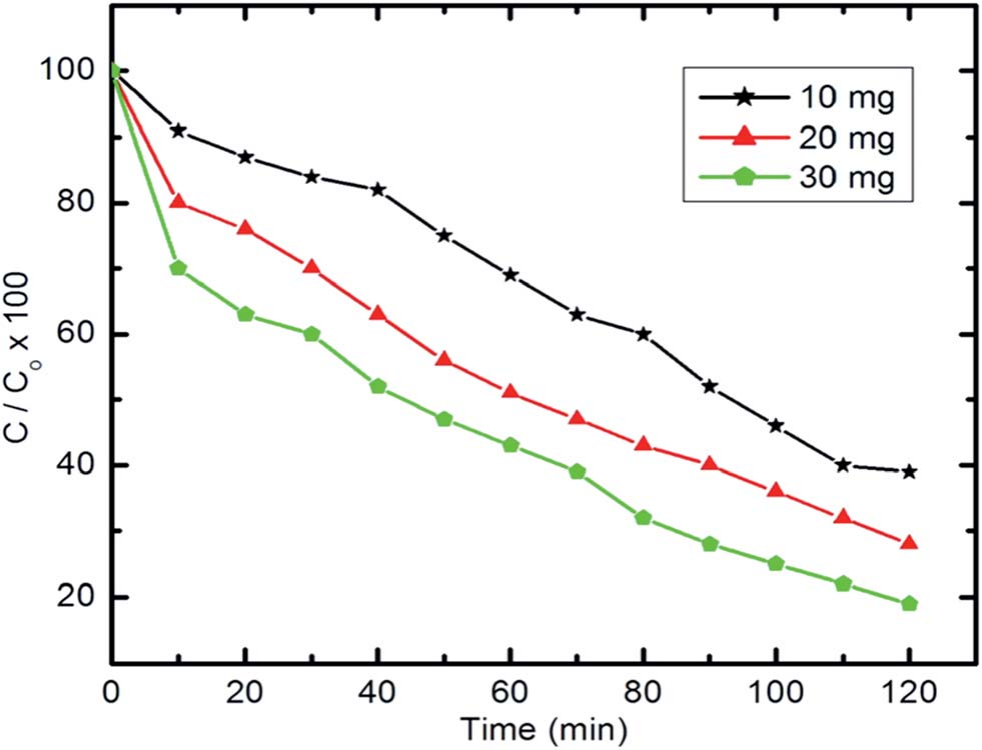
Effect of photocatalyst dose on pyridine degradation efficiency (50 ml Py solution (100 mg l^−1^), 25 °C and pH 7).

The impact of the photocatalyst dose on the efficiency of degradation of pyridine under visible light irradiation was studied by tuning the amount of photocatalyst used between 10 mg and 30 mg at a pyridine concentration of 100 mg l^−1^, as shown in Fig. 13. Our results reveal that upon increasing the dose of catalyst (Co_*x*_Ni_1−*x*_Fe_2_O_4_/SiO_2_/TiO_2_; *x* = 0.9) from 10 to 30 mg in 100 ml of aqueous pyridine, the value of *C*_*t*_/*C*_0_ × 100 decreased from 40 to 19, which shows an increase in degradation efficiency. This direct proportionality between the degradation efficiency and the dose of the reaction medium photocatalyst can be attributed to the increase in the active sites and large surface area available for pyridine degradation.^81^

#### 3.5.3. Effect of pH on degradation efficiency

**Fig. 14:**
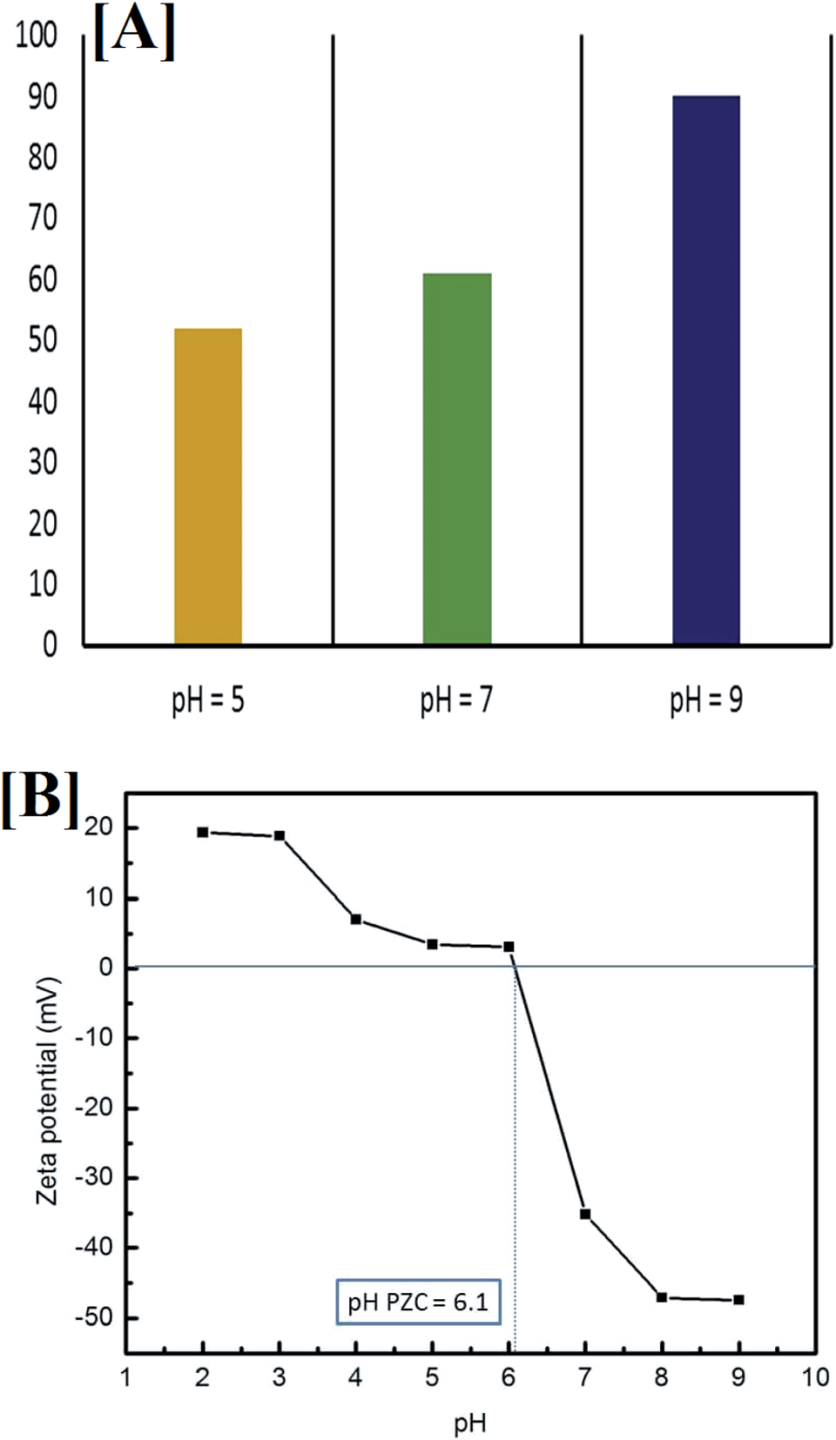
[A] Influence of initial pH on the removal of pyridine (50 ml pyridine (100 mg l^−1^), 10 mg nanocomposite and 100 min irradiation time) and [B] the point of zero charge (PZC) of Co_*x*_Ni_1−*x*_Fe_2_O_4_/SiO_2_/TiO_2_; *x* = 0.9 at different pH values.

The effect of initial pH values of the pyridine solution was studied for 100 min under specified experimental conditions (10 mg of prepared nanocomposite, 50 ml Py solution (100 mg l^−1^), 25 °C). Our results reveal that the optimal pH, which led to degradation of 86.5% of the pyridine, was 9. At pH 5 and 7 degradation efficiencies of only 46.5% and 60.5%, respectively, were recorded. In addition, no increase in pyridine degradation was noticed when the pH was further increased above pH 9, as shown in Fig. 14A, and the degradation efficiency was substantially decreased at pH < 5.

To better understand the relationship between pH and degradation efficiency, the point of zero charge was determined by measuring the zeta potential of the composite at different pH values, from 2 to 10, in the presence of 0.01 M NaCl (electrolyte), as shown in Fig. 14B.

It is clear that when the pH is equal to 7 (neutral media) and when it is more than 7 (alkaline media) the composite exhibited a negative charge (zeta potential = −35.19 mV at pH 7), which stabilized at pH 9 (zeta potential = −47.39). In acidic media (pH less than 7), the composite showed a positive charge, which stabilized at pH 2 (zeta potential = +19.37 mV).

Moreover, the point of zero charge (PZC) was been found to equal 6.1, which is in good agreement with the literature for TiO_2_ NPs (the outer layer of the composite).^82^ It was previously reported that at a pH of more than 8, TiO_2_ NPs are stabilized, with no agglomeration, which is very important for photocatalytic degradation, and this supports the enhanced photocatalytic activity of the prepared composite (with the TiO_2_ outer layer) at pH = 9.^83^ Pyridine is a heterocyclic compound containing nitrogen and, with degradation and upon mineralization, the N atom in the ring is released as ammonia, which can be detected easily by its unpleasant odor.^84^

Generally, it can be proposed that at high values of acidity for the reaction medium, and due to the higher electronegativity of the pyridine N atom compared with the sp^2^ hybridized C atoms in the ring, ammonium salt develops and is released into the solution, which is more stable and leads to a decrease in degradation efficiency. However, in alkaline medium, the anionic state of pyridine supports the absorption of visible light. In addition, the formation of more hydroxyl radicals from hydroxyl ions (OH^−^ → OH˙) in the solution may lead to improvement in the degradation efficiency.^14,85^

#### 3.5.4. Effect of hydrogen peroxide on degradation efficiency

**Fig. 15:**
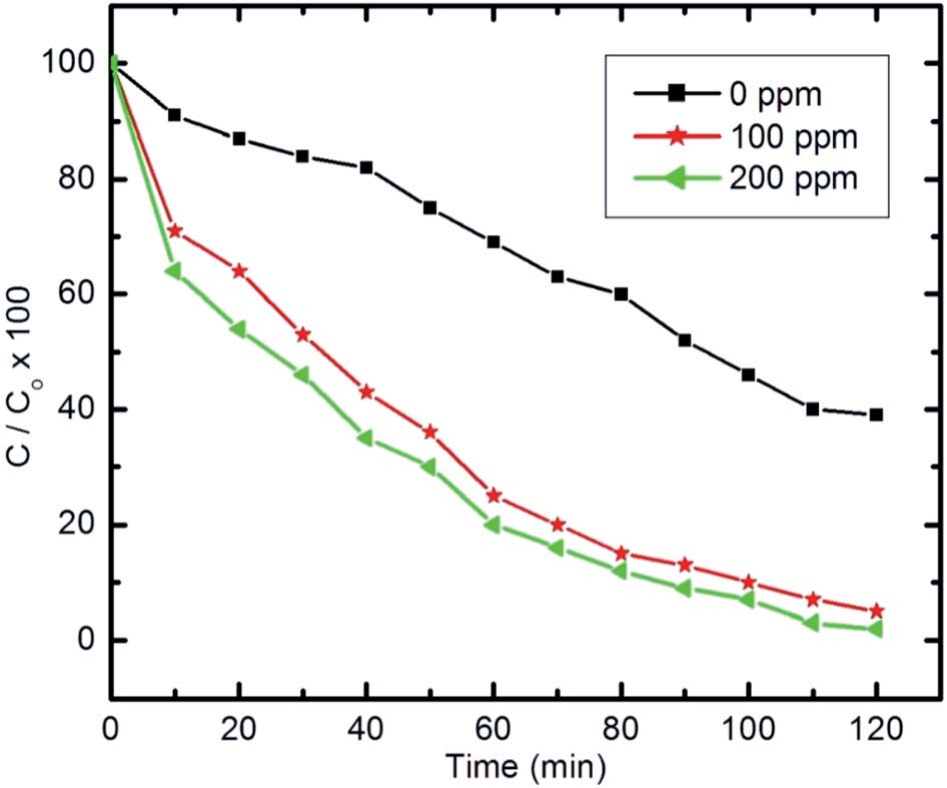
Effect of H_2_O_2_ on pyridine degradation (initial concentration of pyridine *C*_0_ = 100 ppm, 50 ml, 10 mg of nanocomposite and pH 9).

Hydroxyl radicals generated in the pyridine aqueous solution as a result of absorption of visible light (eqn (3)) can form hydrogen peroxide (H_2_O_2_) in the reaction medium. However, increased amounts of hydrogen peroxide may have a critical effect on the total contaminant degradation (eqn (4)). Therefore, a study was performed at different concentrations of H_2_O_2_, as shown in Fig. 15.

Herein, experiments were repeated with pyridine solutions containing H_2_O_2_ to study its effect on the photocatalytic degradation of pyridine (100 mg l^−1^ and 50 ml) with H_2_O_2_ concentrations of 100 ppm and 200 ppm in the solution.

3 H_2_O_2_ + visible light → 2˙OH

4 ˙H + H_2_O_2_ → ˙OH + H_2_O

The amount of H_2_O_2_ that can be formed by the absorbed visible light is too small to dissociate into significant amounts of ˙OH. Therefore, further quantities of H_2_O_2_ are needed to foster the degradation process. Addition of H_2_O_2_ will increase and accelerate the generation of ˙OH in two ways; either through self-decomposition due to the absorption of visible light, or by H_2_O_2_ reduction at the conduction band, as illustrated in eqn (3) and (4), respectively.^14,86^

Generally, to improve the photocatalytic removal of organic compounds, several researchers have investigated the effect of adding external oxidants, such as H_2_O_2_, to improve the process. Under certain conditions, ˙OH is formed by the ready decomposition of H_2_O_2_, as the O–O bond dissociation energy in H_2_O_2_ is only 213 kJ mol^−1^, which is smaller than that of the O–H bond in H_2_O at 418 kJ mol^−1^.^14^ However, after analyzing various studies that suggest the use of H_2_O_2_ to improve the degradation rate of organic pollutants, it is clear that the optimal value for H_2_O_2_ is strongly dependent on the type of organic compound, the equipment configuration and the operating conditions, all of which were identified as having a direct effect on the production rate of ˙OH.

To sum up, it is worth noting that the H_2_O_2_ concentration should not exceed a certain optimal value as it could then recombine with the ˙OH formed, leading to a decrease in the total degradation rate.^14,86,87^

**Fig. 16:**
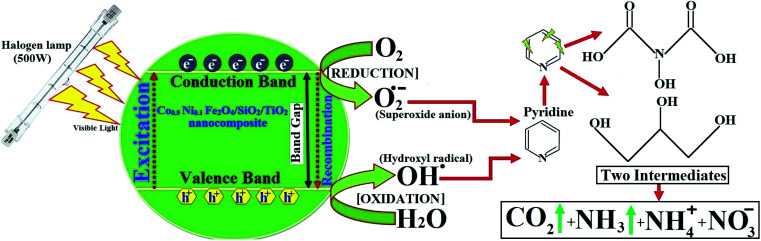
Photocatalyst mechanisms of pyridine using Co_*x*_Ni_1−*x*_Fe_2_O_4_/SiO_2_/TiO_2_; *x* = 0.9 nanocomposite and a possible degraded product.

In the literature on degradation of pyridine and pyridine derivatives there are inconsistencies concerning the reaction mechanism by means of holes or hydroxyl radicals, as shown in Fig. 16. Agrios and Pichat^88^ suggest that pyridine reacts over TiO_2_ predominantly *via* formation of a radical centered on the pyridine ring. Some researchers report that free radicals would be generated by applying visible light simultaneously with oxidants (air bubbling).^89,90^ Wang *et al.*^91^ showed that hydroxyl radicals (˙OH) could be generated indirectly from the application of visible light. Therefore, the degradation relies on the generation of reactive free radicals, especially hydroxyl radicals (˙OH), which is a powerful oxidizing agent having an oxidation potential of 2.33 V, which can undergo rapid and non-selective reaction with most organic and many inorganic solutes.^92^

According to our previous work,^14^ detection of the pyridine intermediate decomposition product by gas chromatography-mass spectrometry (GC-MS) and high-performance liquid chromatography (HPLC) supports a mechanism by means of holes, as shown in Fig. 16, whereas the increase in the degradation rate on bubbling air (O_2_) through the solution evidences the important role played by hydroxyl and superoxide anion (˙O_2_^−^) radicals.

Pyridine wastewater consists of a large amount of molecular H_2_O and ammonia. In addition, N–H⋯O and O–H⋯N intermolecular hydrogen bonds can exist in the wastewater.^93,94^ Molecular H_2_O and ammonia are both polar molecules and can be polarized by visible light in the presence of H_2_O_2_, which causes the dipoles to rotate and line up rapidly (2450 million times per s). Therefore, the frequent pendulum vibration of molecular H_2_O and ammonia leads to breaking and weakening of the intermolecular hydrogen bond between ammonia and H_2_O, which is beneficial for escape of the pyridine decomposition product from the liquid phase to the gas phase (Fig. 16).

In order to confirm the degradation of pyridine, different samples were analyzed by HPLC (direct injection) as mentioned in our previous work.^14^ The results in ref. 14 show the HPLC response of the pyridine sample injection subjected to microwave (MW) radiation for 1 min. The pyridine retention time was 4.435 min. The HPLC results also show the response after 3 min irradiation time for the same sample. The chromatogram shows two other peaks which could be attributed to the pyridine degradation intermediate products according to the above discussion.

Finally, as can be seen in the GC-MS analysis,^14^ the N atom in the pyridine ring is released as NH_3_ upon mineralization and then stripped from solution by aeration.^94^ The ammonium nitrogen (NH_3_–N) is often monitored as a measure of pyridine degradation.^94,95^

## 4 Conclusion

In this work, a recyclable nanocomposite (Co_*x*_Ni_1−*x*_Fe_2_O_4_/SiO_2_/TiO_2_; *x* = 0.9) was designed and fabricated using a layer-by-layer approach. The crystallinity and the effect of gamma radiation on the crystal size of the prepared nanocomposite were identified by XRD, while the average particle size was measured through HRTEM, which revealed the semi-spherical shape of the concentric nanocomposite. The synthesized Co_*x*_Ni_1−*x*_Fe_2_O_4_/SiO_2_/TiO_2_; *x* = 0.9 nanocomposite possesses good purity, as revealed by elemental EDX analysis. The core–multi-shell structure was evaluated using SEM-EDX mapping techniques. The antimicrobial abilities of the nanocomposite, gamma-irradiated nanocomposite and UV-irradiated nanocomposite were then studied. The whole composite was more powerful in terms of its antimicrobial abilities than its separated layers (separate core and two shells). It was active even at low concentrations (10.0 μg ml^−1^) against all tested pathogens. Our results show that the gamma-irradiated Co_*x*_Ni_1−*x*_Fe_2_O_4_/SiO_2_/TiO_2_; *x* = 0.9 nanocomposite was effective against all tested pathogenic microbes as a result of its high surface area and reduction in crystal size due to the gamma rays. The gamma-irradiated nanocomposite exhibited more enhanced antimicrobial abilities than the non-irradiated composite, and this ability increased proportionally with increasing radiation dose (the 100 kGy gamma-irradiated composite was the most active, for example, causing *E. coli* growth inhibition even at 0.024 μg ml^−1^ MIC; Table 2). Biofilm examination also revealed that 100 kGy gamma-irradiated Co_*x*_Ni_1−*x*_Fe_2_O_4_/SiO_2_/TiO_2_; *x* = 0.9 nanocomposite inhibited biofilm formation by 92.82%, 92.29%, and 90.69% against *E. coli*, *C. albicans* and *S. aureus*, respectively. Interestingly, the results for the antimicrobial abilities of UV-irradiated nanocomposite also revealed higher antimicrobial activity of the composite with time, confirming the generation of ROS that can decompose bacterial cells. In addition, SEM-EDX analysis of the treated bacterial cells confirmed the cellular internalization by the nanocomposite in *E. coli* cells and an observable external cell roughness, with subsequent deformation and a decrease in the viable count of *E. coli* cells being observed. Finally, the visible light-assisted photocatalytic degradation of pyridine by the prepared nanocomposite was studied taking into account many parameters that can affect its degradation abilities, such as pyridine initial concentration, photocatalyst dose (nanocomposite dose), different pH values and the presence of H_2_O_2_. The prepared composite showed promising visible light-assisted photodegradation abilities against pyridine. Our work provides a new nanocomposite-based solution for degradation of toxic pollutants and disinfection of water-borne pathogens, which cause serious diseases, and thus offers a new wastewater treatment technique to overcome the problems of global water shortage and water contamination.

## Funding

Not applicable.

## Research involving human participation and/or animals

Not applicable.

## Informed consent

Not applicable.

## Ethical approval

Not applicable.

## Conflicts of interest

The authors declare that they have no conflict of interest.

## Supplementary Material

RA-010-C9RA10505K-s001
