# Supplementary material for: Merits of photocatalytic and antimicrobial applications of gamma-irradiated CoxNi1−xFe2O4/SiO2/TiO2; x = 0.9 nanocomposite for pyridine removal and pathogenic bacteria/fungi disinfection: implication for wastewater treatment
Source: RSC Adv. 2020 Feb 3;10(9):5241–59. doi: 10.1039/c9ra10505k (PMC9049020; doi:10.1039/c9ra10505k)
Supplement: RA-010-C9RA10505K-s001 [file RA-010-C9RA10505K-s001.pdf]

Electronic Supplementary Material (ESI) for RSC Advances.
This journal is © The Royal Society of Chemistry 2020

Supplementary material

Figure S1: Shows image of how $Co_xNi_{1-x}Fe_2O_4$; x=0.9/SiO_2/TiO_2 nanocomposite can be slightly separated from water or medium by an external magnet, the magnetic separation was not so effective due to the relatively-weak core ($Co_{0.9}$ $Ni_{0.1}$ Fe_2O_4 NPs).

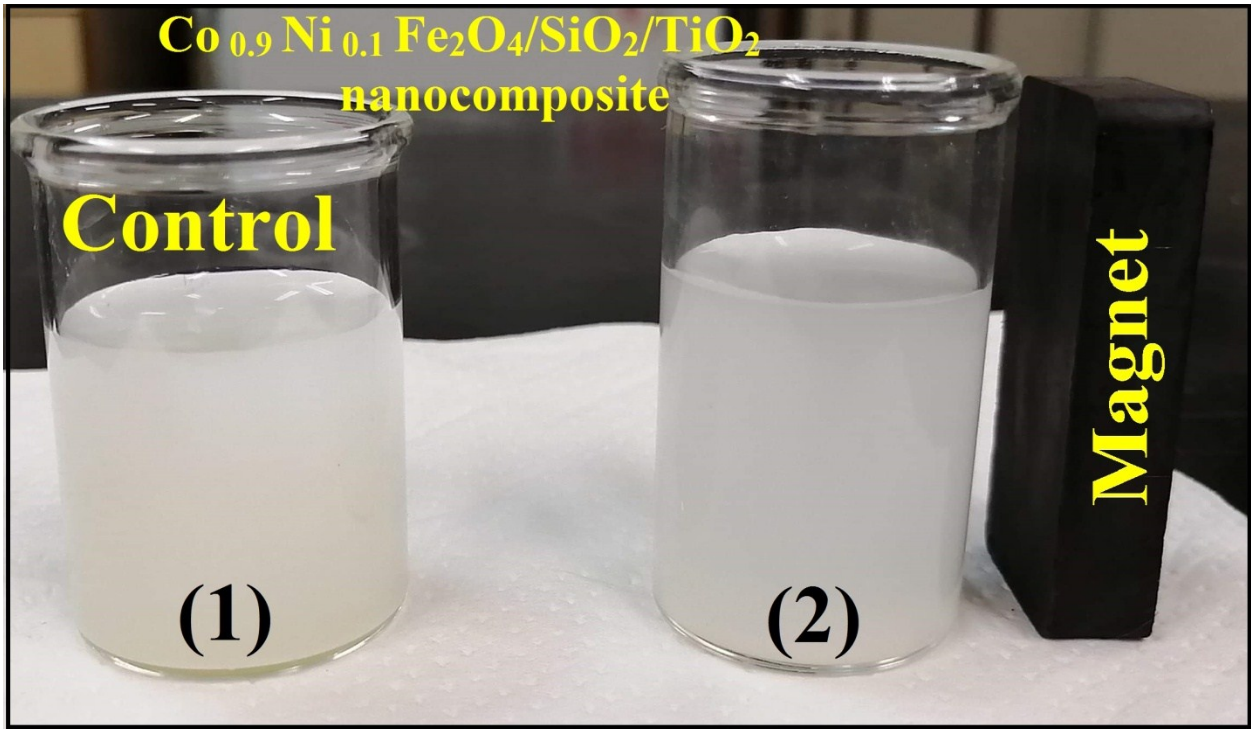

Figure S1: Shows image of how $Co_xNi_{1-x}Fe_2O_4$; x=0.9/SiO_2/TiO_2 nanocomposite can be slightly separated from water or medium by an external magnet, the magnetic separation was not so effective due to the relatively-weak core ($Co_{0.9}$ $Ni_{0.1}$ Fe_2O_4 NPs).
